# Alterations of Purinergic Receptors Levels and Their Involvement in the Glial Cell Morphology in a Pre-Clinical Model of Autism Spectrum Disorders

**DOI:** 10.3390/brainsci13071088

**Published:** 2023-07-18

**Authors:** Lidia Babiec, Anna Wilkaniec, Marta Matuszewska, Ewelina Pałasz, Magdalena Cieślik, Agata Adamczyk

**Affiliations:** Department of Cellular Signalling, Mossakowski Medical Research Institute, Polish Academy of Sciences, Pawińskiego 5, 02-106 Warsaw, Poland; lbabiec@imdik.pan.pl (L.B.); mmatuszewska@imdik.pan.pl (M.M.); epalasz@imdik.pan.pl (E.P.); mcieslik@imdik.pan.pl (M.C.); aadamczyk@imdik.pan.pl (A.A.)

**Keywords:** purinergic signalling, microglia, autism spectrum disorders, valproic acid

## Abstract

Recent data suggest that defects in purinergic signalling are a common denominator of autism spectrum disorders (ASDs), though nothing is known about whether the disorder-related imbalance occurs at the receptor level. In this study, we investigated whether prenatal exposure to valproic acid (VPA) induces changes in purinergic receptor expression in adolescence and whether it corresponds to glial cell activation. Pregnant dams were subjected to an intraperitoneal injection of VPA at embryonic day 12.5. In the hippocampi of adolescent male VPA offspring, we observed an increase in the level of P2X1, with concomitant decreases in P2X7 and P2Y1 receptors. In contrast, in the cortex, the level of P2X1 was significantly reduced. Also, significant increases in cortical P2Y1 and P2Y12 receptors were detected. Additionally, we observed profound alterations in microglial cell numbers and morphology in the cortex of VPA animals, leading to the elevation of pro-inflammatory cytokine expression. The changes in glial cells were partially reduced via a single administration of a non-selective P2 receptor antagonist. These studies show the involvement of purinergic signalling imbalance in the modulation of brain inflammatory response induced via prenatal VPA exposure and may indicate that purinergic receptors are a novel target for pharmacological intervention in ASDs.

## 1. Introduction

Growing evidence demonstrates the involvement of purinergic signalling in brain development and neurotransmission [1,2]. Unlike other neurotransmitter systems, which are characterised by high specificity, the major hallmarks of purinergic signalling are its omnipresence and versatility [3,4]. Not only is ATP secreted from pre- or post-synaptic terminals, as well as from other cells in the central nervous system (CNS) in response to neurotransmitter stimulation, but it is also secreted in response to a variety of other stimuli, like inflammation or cellular damage [5,6]. Moreover, the release of ATP is mediated via various mechanisms, including vesicle exocytosis, depending on the vesicular nucleotide transporter (VNUT) and SNARE complex [7]; the activation of membrane channels (such as connexins, pannexins, P2X7 receptor); or specific transporters [8,9,10,11], like ATP-binding cassette (ABC) transporter [8,9,10]. Unlike classical neurotransmitters, which are inactivated via their reuptake from the synaptic cleft, ATP undergoes rapid enzymatic degradation by cell membrane ectonucleotidases [12]. Each of the products (ATP, ADP, AMP, adenosine) involved in the multistep enzymatic conversion of ATP to adenosine can activate different types of purinergic receptors localized on pre- and post-synaptic membranes [13]. Currently, four receptors of adenosine (P1 type), seven subtypes of ionotropic ATP receptors (P2X), and eight subtypes of metabotropic (P2Y) ATP receptors are recognized, many of which can form heteromultimers [14]. ATP and its end product, adenosine, are important signalling molecules in both pre-natal and post-natal development [15], regulating the short-term neurotransmission/neuromodulation in the CNS, as well as longer-term effects, including neuronal development and neuroregeneration [14,16,17,18]. Apart from neurons, purinergic receptors are also present in glial cells, as well as in each of the following cell subtypes: microglia, astrocytes, and oligodendrocytes. These subtypes express a unique pattern of purinergic receptors, which are responsible for orchestrating specific intracellular downstream signalling that leads to specific responses [19]. Moreover, purinergic receptors are indispensable in regulating the specific interaction between glia and neurons that largely extend beyond synapses [3]. Neuronal release of ATP and adenosine during action potential firing was previously shown to evoke calcium waves in astrocytes, Schwann cells, and oligodendrocytes, as well as state the novel mechanism of sensing neuronal activity by glial cells [20,21]. ATP also regulates the activity of microglia, which are the immune cells of the nervous system, leading to rapid changes in their morphology and migration [22,23], as well as triggering cytokine secretion [24]. Glial cells not only form “the scaffold” for neurons but also participate in the synaptogenesis and synaptic plasticity, homeostasis, elimination of used transmitters, facilitation of signal transduction; and in immune system responses [25]. Importantly, astrocytes and microglia strictly cooperate, and purinergic signalling is the main mode of communication between them [3,26,27,28]. Thus, ATP and adenosine may be indispensable in bidirectional microglia–neuron communication, as they enable glia to detect synaptic function, further propagate the signal through the glial cell network, and influence synaptic function at distant sites. These unique properties greatly expand the significance of purinergic signalling in brain function, especially under pathological conditions accompanying neurodegenerative or neurodevelopmental disorders [29,30]. Many previous data have found defects in nucleoside/nucleotide signalling to be a common denominator of autism spectrum disorders (ASDs). Studies of ASD animal models have indicated that the deregulation of the enzyme activities involved in the control of ATP and adenosine levels may be involved in the impairment of social interaction [31]. Some clinical reports point out that glial cell deregulation is also engaged in pathological processes that may lead to autism since their critical role in enabling brain functions, astrocytes, and microglia are increasingly linked to ASD features [32]. To date, although astrocytes and microglia actively respond to brain homeostasis aberration via activation, which is reflected in morphology change [33,34], most studies on ASD have focused on changes in the number of astroglia markers or the number of glial cells, rather than a thorough analysis of morphological changes in these cells [35]. Recent studies indicate that purine metabolism in glial cells seems to be the most strikingly disturbed pathway in autism [36], though, currently, nothing is known about whether the ASD-related imbalance also occurs at the receptor level. Moreover, despite the detailed role of glial cell function in ASD, little is known about the involvement of particular purinergic receptor subtypes in abnormalities of these cells. Given that different purinergic receptor subtypes may participate in various processes across CNS development, in the present study, we first aimed to investigate the changes in the expression pattern of the selected purinergic receptors in the cortex and hippocampus of rats at three developmental stages: the late prenatal stage (gestational day 19), weanling (postnatal day 25), and adolescence (postnatal day 52). Next, we analysed whether, in the brain of adolescent male autistic-like rats, structure-dependent alterations in particular purinergic receptors’ expression in the brain occur. In addition, we explore whether changes in purinergic signalling may be associated with the activation of glial cells and neuroinflammatory responses.

## 2. Materials and Methods

### 2.1. Ethical Statement

All experiments conducted with animals were approved by the Local Ethics Committee for Animal Experimentation in Warsaw, Poland (reference numbers: WAW2/148/2018, WAW2/036/2019, WAW2/128/2021) and were carried out following the EC Council Directive of 24 November 1986 (86/609/EEC), the ARRIVE guidelines, and guidelines published in the NIH Guide for the Care and Use of Laboratory Animals. Additionally, the principles presented in the “Guidelines for the Use of Animals in Neuroscience Research” by the Society for Neuroscience were followed. We made an effort to use the minimal number of animals necessary to obtain results and proceeded with the animals gently to reduce stress and suffering.

### 2.2. Animals—In Vivo Model of ASD

We obtained Wistar female rats from the Animal House of the Mossakowski Medical Research Institute, Polish Academy of Sciences (Warsaw, Poland). The rats were aged between 12 and 15 weeks and had a standard weight range of 210–250 g. They were bred according to the SPF standard for small rodents and kept under a 12-h light/dark cycle with access to food and water ad libitum, and the temperature and humidity were strictly controlled. 

To determine pregnancy, the presence of a vaginal plug was observed and recorded as embryonic day 0. On gestational day 12.5, pregnant rats received a single intraperitoneal injection of valproic acid (VPA) at a dose of 450 mg/kg of body weight to induce the ASD phenotype [37]. Control dams were given a single injection of saline. All pregnant dams were injected at the same time of the day (noon). To minimize the risk of litter effect, randomly selected animals from at least 2 or 3 litters in each experimental group. 

For purinergic receptors analysis, several of the control and VPA dams were sacrificed on embryonic day 19, and foetal brain samples (cerebral cortices with hippocampi) were microdissected. The remaining pregnant rats were allowed to give birth and raise offspring under normal conditions. The day of birth was considered postnatal day (PND) 1. At 22–23 days of age, the rat pups were separated from their mothers and housed in groups of 3–4 individuals in open polycarbonate cages with an enriched environment. Juvenile and adolescent rats (25 PND and 52 PND, respectively) were sacrificed by decapitation. The brains were quickly removed, and two brain parts, the hippocampus and cerebral cortex, were collected and placed in liquid nitrogen.

**Scheme 1:**
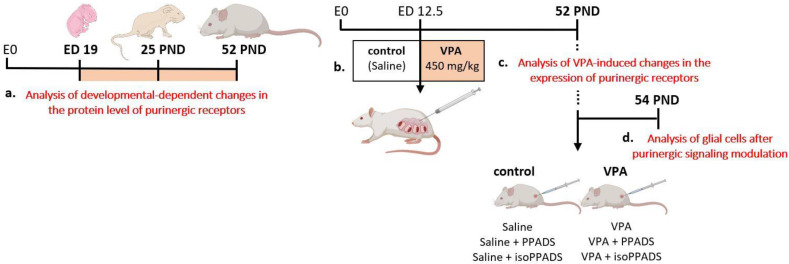
Experimental design. (**a**) The analysis of changes in the levels of specific purinergic receptors during rat brain development was conducted at three stages: ED 19, 25 PND, and 52 PND. (**b**) A Valproic acid-induced model of ASD was performed by a single intraperitoneal injection of VPA (450 mg/kg, i.p.) or saline into pregnant rats. (**c**) The investigation of VPA-induced alteration on the expression of selected purinergic receptors was performed on the brains of 52-day-old animals. (**d**) To analyse the modulation of purinergic signalling, rats at 52 PND were divided into three groups and subsequently injected with PPADS (P2X receptor antagonist), isoPPADS (12.5 mg/kg, i.p.) (P2 receptor antagonist), or saline. Analysis of glial cells and inflammatory cytokine expression was performed on the brains of 54-day-old animals. The figure was created using BioRender.com.

To analyse the modulation of purinergic signalling, male offspring from the control or VPA groups were divided into three groups at 52 PND. The rats received a single intraperitoneal injection of either PPADS or isoPPADS at a dose of 12.5 mg/kg of body weight. Control rats received the appropriate volume of the vehicle. After 48 h, the rats were sacrificed. Samples for biochemical analysis were collected as mentioned earlier, while for IHC staining, rats were perfused with 4% PFA before decapitation and brain removal. The samples were stored at −80 °C until use. Experimental design is shown on the Scheme 1.

### 2.3. Quantitative Real-Time Polymerase Chain Reaction (qRT-PCR)

To isolate RNA from the brain samples, we used TRI-reagent (Sigma-Aldrich, St. Louis, MO, USA). The extraction procedure was carried out according to the manual provided by the manufacturer. RNA quantity and quality were analysed with spectrophotometer measurements using NanoDrop ND-1000 (NanoDrop Technologies, Wilmington, DE, USA). Subsequently, we digested the remaining trace amounts of DNA with DNase I (Sigma-Aldrich, St. Louis, MO, USA) following the manufacturer’s instructions. 

For reverse transcription, we utilized the High Capacity cDNA Reverse Transcription Kit (Applied Biosystems, Foster City, CA, USA) in accordance with the manufacturer’s instructions. The mRNA level of the selected purinergic receptors was measured using real-time PCR, employing the TaqMan Gene Expression Assays (Applied Biosystems, Foster City, CA, USA) listed in Table 1. The analysis was performed on the ABI PRISM 7500 apparatus. Actb was used as the reference gene, and each sample was analysed in three technical replicates. The ΔΔCt method was employed to calculate the relative changes in mRNA levels, and the results were expressed as RQ.

### 2.4. Western Blot Analysis

To determine the protein level of selected purinergic receptors, we employed the Western blotting method. After homogenising the tissue samples, we mixed them with a Laemmli buffer. Denaturation was carried out at 95 °C for 5 min. Proteins were separated using standard 10% or 15% SDS-PAGE gels. Subsequently, the proteins were transferred to nitrocellulose membranes using the “wet” transfer method at 50 V for 2 h.

Immunodetection of specific antibodies began with a 5-min wash in TBS-T (Tris-buffered saline with Tween 20 buffer: 100 mM Tris, 140 mM NaCl, and 0.1% Tween 20, pH 7.6). To block non-specific binding, we incubated the membranes with a 5% BSA solution in TBS-T or a 5% non-fat milk solution in TBS-T for 1 h at room temperature (RT). The membranes were then incubated with the primary antibodies, and detailed protocols can be found in Table 2. After three washes with TBS-T, the membranes were incubated with appropriate secondary antibodies for 1 h at RT, followed by another 3 washes with TBS-T. 

The detection of antibodies was performed using a chemiluminescent reaction and the Clarity Western ECL Substrate (Bio-Rad Laboratories, Hercules, CA, USA) according to the manufacturer’s instructions. After stripping, the immunolabelling of GAPDH as a loading control was performed. Densitometric analysis was conducted using TotalLab v1.11 software.

### 2.5. Immunohistochemistry Staining

The animals were deeply anaesthetized with ketamine and transcardially perfused with cold (4 °C) phosphate-buffered saline (PBS), followed by 4% paraformaldehyde (PVA) in PBS. The brains were then removed and placed in 4% PVA for 24 h, followed by immersion in 30% sucrose for cryoprotection. The brains were frozen on dry ice and stored at −80 °C. 

For double immunohistochemical staining, the animal brains from the control and VPA groups at 52 PND were used, while for single staining, the animal brains from the control, control + PPADS, control + isoPPADS, VPA, VPA + PPADS, and VPA + isoPPADS groups at 54 PND were used. Staining was performed on 40 µm coronal sections of the hippocampus and cerebral cortex.

For double staining, the sections were first incubated with primary antibodies against MAP-2, GFAP, or Iba-1 for 1 h at RT and overnight at 4 °C. On the following day, the sections were washed in PBS (3 × 5 min) and incubated with the following primary antibodies: P2X1 and P2Y1. Then, they were directly labelled with an ATTO-488 fluorescent dye, or P2X7 and P2Y12. For single staining, primary antibodies against Iba-1 and GFAP were used. 

The next day, the sections were washed in PBS (3 × 5 min), and incubated for 1 hour at RT with the appropriate secondary antibody conjugated with Alexa Fluor 594. The secondary antibody was diluted to working concentrations in PBS with 1% BSA, and 0.3% Triton X100. To prevent photobleaching, incubation with fluorescent antibodies was conducted in the dark. The concentration and conditions of the antibodies are indicated in Table 3. Finally, after washing (3 × PBS), the sections were transferred onto microslides. The slides were coated with Vectashield Vibrance mounting medium with DAPI, which counterstained cell nuclei, and covered with a coverslip.

Images were obtained at the Laboratory of Advanced Microscopy Techniques, Mossakowski Medical Research Institute, Polish Academy of Sciences, using a confocal laser scanning microscope LSM780 (Zeiss, Jena, Germany) with z-stack acquisition using a 10× objective. For the analysis of glial cell morphology, a 40× objective was utilized, as described in [38].

Quantitative analysis of astrocytic and microglial processes, cell bodies, and Sholl analysis were performed following the method outlined by Sanagi et al. [39] with modifications. Five microglia or astrocytes were randomly selected per section, and their values were averaged, except for astrocytes in the hippocampus, for which we averaged five cells derived from each of the three hippocampal layers. 

To analyse the images of astrocytes or microglia, the images were converted to an 8-bit format, and the dendrites of individual cells were traced using the NeuronJ plugin for Fiji [40]. The surface area of the cell body, the surface area of the dendritic tree, the number of primary dendrites (those originating from the cell soma), the number of secondary dendrites, and the total length of processes belonging to each glial cell were analysed. 

The branches of individual glial cells were determined using the Sholl method [41]. Each cell was analysed by selecting the centre of its body, and then the number of intersections at circles of increasing diameter from the centre was counted using the SNT plugin for Fiji. The diameters of all processes were measured at intervals of 2.5 μm.

### 2.6. Statistical Analysis

All results are presented as the mean values ± standard deviation (SD). To analyse differences between the average values, we used a Student’s *t*-test (between two groups) or one-way analysis of variance (ANOVA) with a Bonferroni comparison post hoc test (between multiple groups). Statistical significance was considered at *p* < 0.05. The analysis was conducted using Graph Pad Prism version 6.0 (Graph Pad Software, San Diego, CA, USA).

## 3. Results

### 3.1. The Expression of Selected Purinergic Receptors during Brain Development

To gain insight into the changes in purinergic receptor expression at different stages of central nervous system (CNS) development, the protein levels of specific receptors were evaluated in the cortex with hippocampus isolated from the foetal brain (ED19), as well as in the cerebral cortex and hippocampus isolated from 25-day-old (weanling, PND 25), and 52-day-old (young adult, PND 52) rats. An average of four animals from two litters were used for each data point.

**Figure 1:**
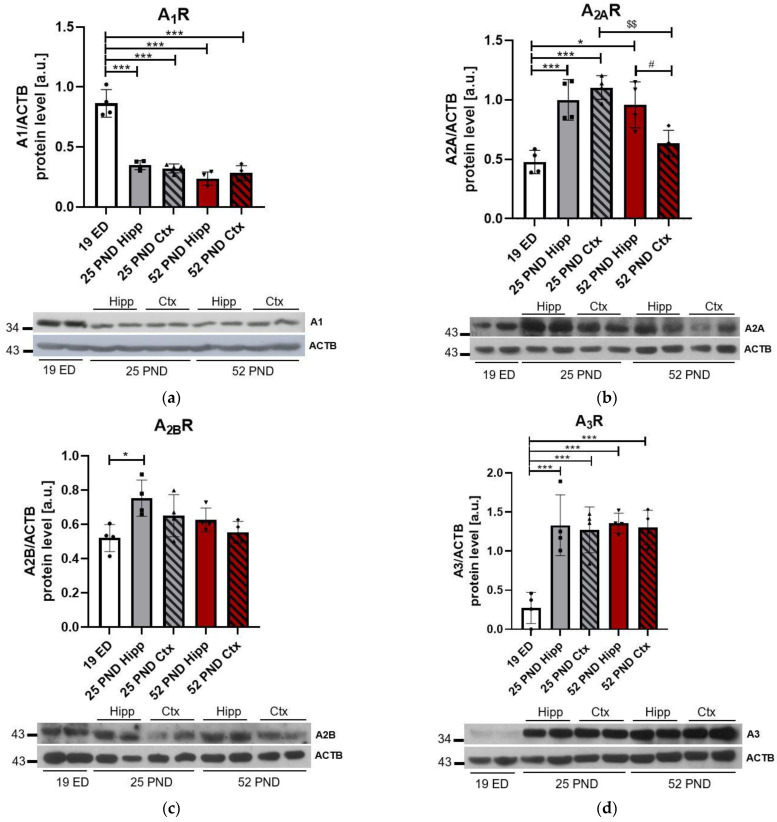
Protein expression of adenosine receptors during rat brain development. The immunoreactivity of A1 (**a**), A2a (**b**), A2b (**c**), and A3 (**d**) receptors in the brain of 19-day-old rat embryos, as well as in the hippocampus (Hipp) and cortex (Ctx) of 25-day-old and 52-day-old rats, was determined using Western blotting. Actin (ACTB) was used as a loading control. The data represent the mean value ± SD from four independent experiments. * *p* < 0.05, *** *p* < 0.001 vs. ED19, $$ *p* < 0.01 vs. cortex PND 25, # *p* < 0.05 vs. hippocampus PND 52, as determined using one-way ANOVA followed by the Bonferroni post hoc test.

It was observed that all subtypes of adenosine receptors were present in the embryonic brain, albeit to varying extents, and their expression continued into adulthood (Figure 1). The A1 receptor showed strong expression in the embryonic brain, with a subsequent decline in expression (Figure 1a). The expression of the A2a receptor was lowest at ED19 and significantly increased in the cortex and hippocampus of rats at PND 25, as well as in the hippocampus at PND 52 (Figure 1b). In adulthood, the level of the A2a receptor significantly decreased in the brain cortex compared to the hippocampus (Figure 1b). Furthermore, the A2a receptor was significantly upregulated in the brain cortex of weanling animals compared to young adult rats. This may be related to the important role of A2a receptors in controlling glutamatergic long-term potentiation (LTP) [42] during the enhanced synaptic plasticity observed during the weanling period in rats [43]. The level of the A2b receptor for adenosine remained constant thorough the investigated developmental stages, with a slight increase observed in the hippocampus of 25-day-old animals (Figure 1c). The A3 receptor level was lowest in the embryonic brain but significantly increased at PND 25 and remained constant until adulthood (Figure 1d).

**Figure 2:**
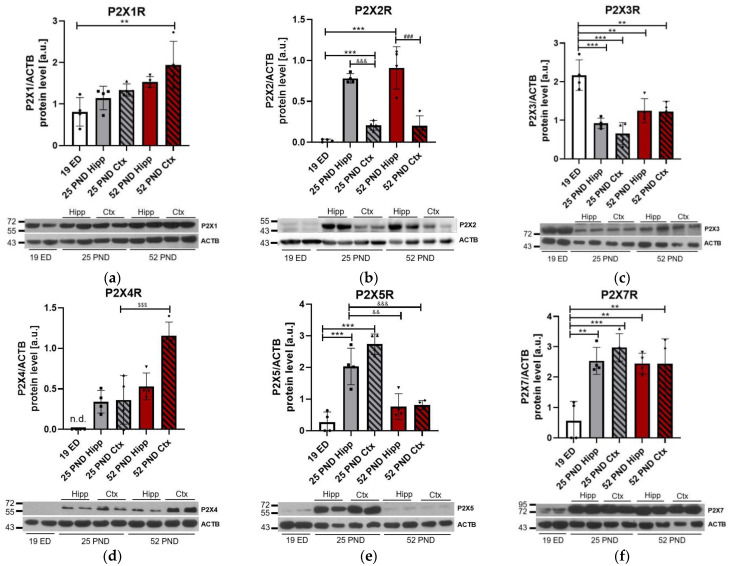
Ionotropic purinergic receptor protein expression during rat brain development. The immunoreactivity of P2X1 (**a**), P2X2 (**b**), P2X3 (**c**), P2X4 (**d**), P2X5 (**e**), and P2X7 (**f**) receptors in the brain of 19-day-old rat embryos, as well as in the hippocampus (Hipp) and cortex (Ctx) of 25- and 52-day-old rats, was determined using Western blotting. Actin (ACTB) was used as a loading control. The data represent the mean value ± SD from four independent experiments. ** *p* < 0.01, *** *p* < 0.001 vs. ED19, && *p* < 0.01, &&& *p* < 0.001 vs. hippocampus PND 25, $$$ *p* < 0.001 vs. cortex PND 25, ### *p* < 0.001 vs. hippocampus PND 52, as determined using one-way ANOVA followed by the Bonferroni post hoc test; n.d.—not detected.

From the investigated ionotropic receptors for ATP, the level of the P2X1 receptor remained constant throughout rat brain development, with a minor increase observed in the cortex of 52-day-old animals (Figure 2a). The P2X2 receptor exhibited the lowest expression in the embryonic brain, and its immunoreactivity significantly increased in the hippocampus of 25-day-old and young adult animals. P2X2 receptor was also significantly upregulated in the hippocampi of both weanling and young adult animals when compared to cortexes at their respective ages (Figure 2b). A marked predominance of the P2X3 receptor was observed in the foetal brain, followed by a substantial decline during postnatal periods (Figure 2c). In contrast, the P2X4 receptor was not detected at ED19, but its expression was observed in the brains of weanling rats, with the highest level observed in the cortex of 52-day-old animals (Figure 2d). 

The P2X5 and P2X7 proteins exhibited the lowest expression at ED19. While a significant elevation in the P2X5 receptor expression level was observed in both the hippocampus and cortex of weanling animals, the protein expression of this receptor significantly declined at PND 52 (Figure 2e). The functional role of the P2X5 receptor in the CNS has been previously demonstrated to be widely distributed in adult rodents [44], in contrast to humans, where it occurs as a nonfunctional splice variant [45]. Therefore, the peak expression of this receptor during weaning may suggest its important role during brain maturation. Interestingly, several reports have linked P2X5 receptor expression with the differentiation and turnover of various non-neuronal cells, as well as in different cancer tissues [46]. However, to date, no data are available on the functional role of this receptor in the CNS. 

On the other hand, the P2X7 receptor was found to be abundantly present in the brain tissue of both 25-day-old and young adult animals (Figure 2f).

**Figure 3:**
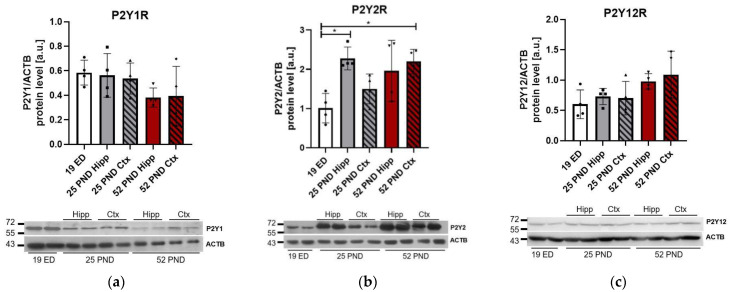
Metabotropic purinergic receptor protein expression during rat brain development. The immunoreactivity of P2Y1 (**a**), P2Y2 (**b**), and P2Y12 (**c**) receptors in the brain of 19-day-old rat embryos, as well as in the hippocampus (Hipp) and cortex (Ctx) of 25- and 52-day-old rats, was determined using Western blotting. Actin (ACTB) was used as a loading control. The data represent the mean value ± SD from four independent experiments. * *p* ˂ 0.05 vs. ED19, as determined using one-way ANOVA followed by the Bonferroni post hoc test.

From the large group of metabotropic purinergic receptors, we selected P2Y1, P2Y2, and P2Y12 for our investigations based on their role in synaptic transmission, regulation of voltage-gated ion channels, modulation of neuronal circuits, gliotransmission, regulation of inflammatory response, and synaptic plasticity [47]. Moreover, those receptors have also been shown to regulate the release of neurotransmitters, including glutamate, in the synapses of the hippocampus and cerebral cortex [48], as well as to inhibit the excitatory transmission mediated by postsynaptic NMDA receptors and to increase the inhibitory action of the GABA receptors, promoting LTP [47,49]. We observed that all investigated P2Y receptor subtypes were expressed in the embryonic tissue. The levels of both P2Y1 and P2Y12 receptors remained constant in all investigated developmental periods (Figure 3a,c). In contrast, the level of the P2Y2 receptor slightly increased in the hippocampus of 25-day-old animals, as well as in the cortex of 52-day-old rats (Figure 3b).

### 3.2. The Impact of Prenatal Exposure to Valproic Acid (VPA) on the mRNA, Protein Expression Patterns, and Immunolocalization of Purinergic Receptors Subtypes in the Brain of 52-Day-Old Animals

Prenatal exposure to VPA produces relevant autistic-like phenotypes in rat offspring, and the specific implementation of this model was recently validated in our study to induce pathological alterations in synapse ultrastructure and function, as well as to cause aberrant behavioural outcomes in offspring [50]. Using this model, we have previously demonstrated the disturbances in purinoreceptors levels occurring at the prenatal stage in animals subjected to VPA treatment [51]. Additionally, the activity of selected purinergic receptor subtypes was significantly altered in neuronal cells isolated from embryonic tissue, suggesting their strong involvement in improper brain development during the early stage of brain formation. In this study, we investigated whether significant alteration in purinergic receptor levels also occurs during the developmental period of late adolescence (PND 52), a critical phase for the exacerbation of autism-related symptoms.

Pregnant rats were injected with VPA or saline (control) during midgestation (ED12.5), and cortexes and hippocampi from the offspring at PND 52 were collected. The mRNA levels and protein expression of selected P1, P2X, and P2Y receptors were measured as described above and compared to the matched control samples. Five to six animals from at least three litters were used. Average values were compared between saline and VPA offspring for each receptor in the selected brain regions. For immunohistochemistry staining, we investigated the abundance of only those receptors in which we observed the most prominent changes in the brains of animals prenatally exposed to VPA. Three animals from at least two litters were included for analysis. Images from the brains of VPA offspring were analysed and compared to control samples for each selected receptor in the prefrontal cortex and hippocampus of 52-day-old animals.

**Figure 4:**
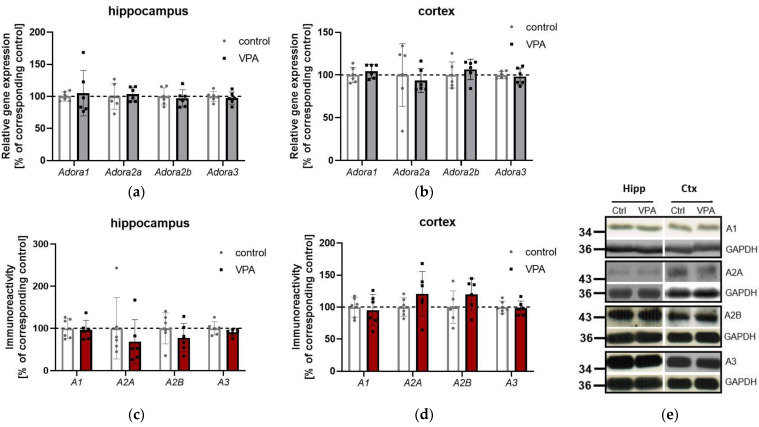
The effect of prenatal treatment with VPA on the gene expression and protein levels of adenosine receptors in the hippocampus and cortex of 52-day-old rats. The mRNA levels of adenosine receptors in the hippocampus (**a**) and cortex (**b**) of young adult animals prenatally exposed to VPA treatment were measured by real-time PCR and normalized to Actb (β-actin). Data are shown as a percentage of corresponding control and represent the mean value ± SD for 5–6 independent experiments. The immunoreactivity of adenosine receptors in the hippocampus (**c**) and cortex (**d**) of young adult animals prenatally exposed to VPA treatment was analysed by Western blotting. Glyceraldehyde-3-phosphate dehydrogenase (GAPDH) was used as a loading control. Representative pictures are shown (**e**). Data are shown as a percentage of the corresponding control and represent the mean value ± SD for 5–6 independent experiments. Statistical analysis was determined using the Student’s *t*-test.

We analysed the expression of adenosine receptors in the brains of animals prenatally exposed to VPA. As shown in Figure 4, the mRNA levels for adenosine receptors in both the hippocampus (Figure 4a) and cortex (Figure 4b) did not change after VPA prenatal exposure compared to the control. Additionally, the analysis of protein levels in the investigated brain structures showed no alterations (Figure 4c,d).

**Figure 5:**
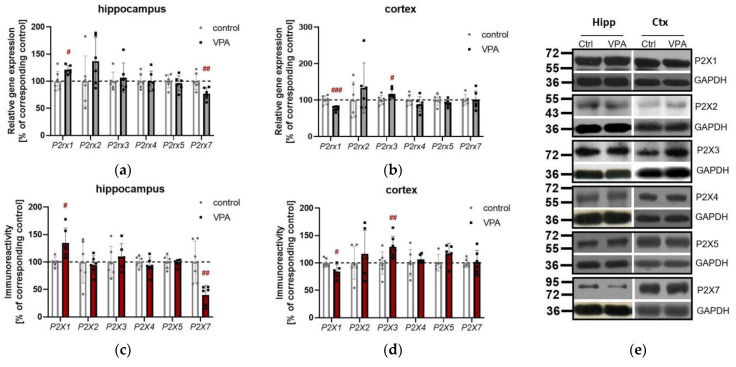

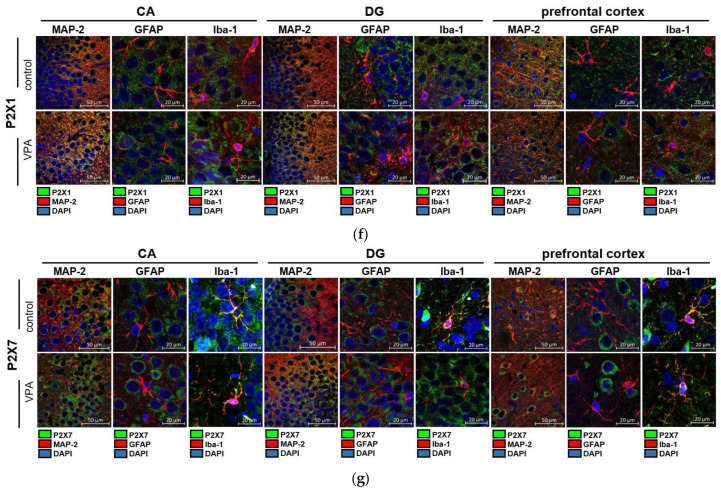
The effect of prenatal treatment with VPA on the gene expression, protein level, and immunolocalization of selected ionotropic receptors in the hippocampus and cortex of 52-day-old rats. The mRNA levels of adenosine receptors in the hippocampus (**a**) and cortex (**b**) of young adult animals prenatally exposed to VPA treatment were measured by real-time PCR and normalized to Actb (β-actin). Data are shown as a percentage of the corresponding control and represent the mean value ± SD for 5–6 independent experiments; # *p* ˂ 0.05, ## *p* < 0.01, ### *p* < 0.001 vs. corresponding control, as determined using the Student’s *t*-test. The immunoreactivity of ionotropic receptors in the hippocampus (**c**) and cortex (**d**) of young adult animals prenatally exposed to VPA treatment was analysed by Western blotting. Glyceraldehyde-3-phosphate dehydrogenase (GAPDH) was used as a loading control. Representative pictures are shown (**e**). Data are presented as a percentage of corresponding control and represent the mean value ± SD for 5–6 independent experiments; # *p* ˂ 0.05, ## *p* < 0.01, ### *p* < 0.001 vs. corresponding control, as determined using the Student’s *t*-test. The immunohistochemical analysis of co-localisation of the P2X1 receptor (green) (**f**) in the neurons (MAP-2, red), astrocytes (GFAP, red) and the glial cells (Iba-1, red) (**f**), as well as the analysis of co-localisation of the P2X7 receptor (green) (**g**) in the neurons (MAP-2, red), astrocytes (GFAP, red), and the glial cells (Iba-1, red) (**g**), was determined in the cortex, dentate gyrus (DG), and cornu ammonis (CA) of the hippocampus. The nuclei were counterstained with DAPI (blue). Scale bar = 50 μm for MAP-2 and 20 μm for GFAP and Iba-1.

The expression of ionotropic receptors in the brain, caused by prenatal VPA treatment, was distinct and region-specific (Figure 5). In the hippocampus, the P2rx1 mRNA significantly increased, whereas the mRNA level of P2rx7 was lower in this brain structure at PND 52 (Figure 5a). These results are consistent with the analysis of protein levels, where we detected an increase in P2X1 and a significant decrease in P2X7 (Figure 5c). In contrast, in the cortex of animals prenatally exposed to VPA, the mRNA level of the ionotropic P2rx1 decreased, and the P2rx7 remained unchanged, but there was an increase in the expression of P2rx3 (Figure 5b). The significant decrease in P2X1 and the elevation of the P2X3 receptor in the cortex of young adult VPA rats were also confirmed at the protein level (Figure 5d). Prenatal exposure to VPA treatment did not influence the expression and protein levels of P2X2, P2X4 and P2X5 receptors in the analysed brain structures (Figure 5a–d). In the following studies, based on the previous data concerning the relevance of particular ionotropic purinergic receptors to brain function [52,53], we also analysed the co-localization of P2X1 and P2X7 receptors on neurons and microglial cells (the nonoverlapping images for the particular photograph are included in Supplementary Figures S1–S6). As shown in Figure 5f, the P2X1 (green immunostaining) co-localized predominantly with neuronal MAP-2-positive cells of either the prefrontal cortex or DG and CA regions of the hippocampus. Basal P2X1 expression in the GFAP-stained astrocytes in the prefrontal cortex and hippocampal areas was low, whereas positive co-expression of the P2X1 with the glial marker Iba-1 was not visible. Prenatal treatment with VPA produced a noticeable increase in P2X1 immunoreactivity in the neuronal cells in the hippocampal areas, while P2X1 neuronal staining was visually decreased in the prefrontal cortex. We did not observe any VPA-induced changes in P2X1 expression in GFAP-stained astrocytes.

The immunohistochemical staining of P2X7 revealed that this receptor is abundant in the perikarya of granule cells in the CA and DG regions of the hippocampus, as well as in the neurons of the prefrontal cortex. The immunostaining of this receptor was also observed in the microglial processes, but it was not visualized on the astrocytes. In both regions of the hippocampus examined, the immunostaining of this receptor visually decreased in the microglial cells of animals treated with VPA, but its abundance on neuronal cells appeared unchanged in the investigated brain areas (Figure 5g).

**Figure 6:**
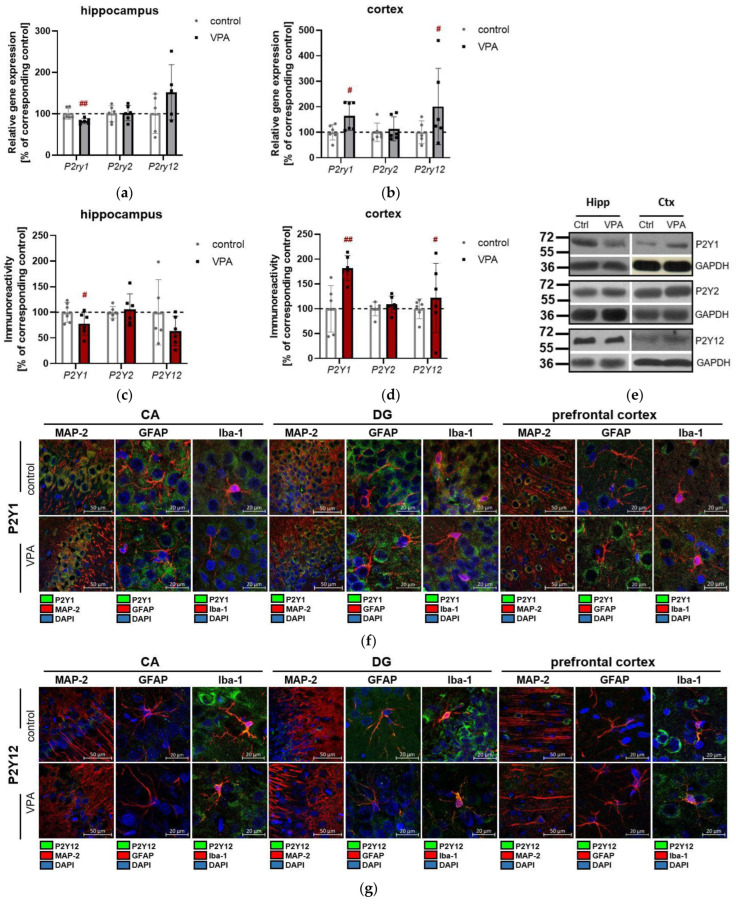
The effect of prenatal treatment with VPA on the gene expression, protein level, and immunolocalization of selected metabotropic receptors in the hippocampus and cortex of 52-day-old rats. The mRNA levels of metabotropic receptors in the hippocampus (**a**) and cortex (**b**) of young adult animals prenatally exposed to VPA treatment were measured by real-time PCR and normalized to Actb (β-actin). Data are shown as a percentage of the corresponding control and represent the mean value ± SD for 5–6 independent experiments; # *p* ˂ 0.05, ## *p* < 0.01 vs. corresponding control as determined using the Student’s *t*-test. The immunoreactivity of metabotropic receptors in the hippocampus (**c**) and cortex (**d**) of young adult animals prenatally exposed to VPA treatment was analysed by Western blotting. Glyceraldehyde-3-phosphate dehydrogenase (GAPDH) was used as a loading control. Representative pictures are shown (**e**). Data are presented as a percentage of the corresponding control and represent the mean value ± SD for 5–6 independent experiments; # *p* ˂ 0.05, ## *p* < 0.01 vs. corresponding control as determined using the Student’s *t*-test. The immunohistochemical analysis of co-localisation of the P2Y1 receptor (green) (**f**) in the neurons (MAP-2, red), astrocytes (GFAP, red) and the glial cells (Iba-1, red) (**f**), as well as the analysis of co-localisation of the P2Y12 receptor (green) (**g**) in the neurons (MAP-2, red), astrocytes (GFAP, red), and the glial cells (Iba-1, red) (**g**), was determined in the cortex, dentate gyrus (DG), and cornu ammonis (CA) of the hippocampus. The nuclei were counterstained with DAPI (blue). Scale bar = 50 μm for MAP-2 and 20 μm for GFAP and Iba-1.

Regarding the metabotropic receptors, we observed a significant decrease in the mRNA level of P2ry1 in the hippocampus of young adult rats following VPA exposure (Figure 6a), which was confirmed by the lower protein level for this receptor (Figure 6c). In the cerebral cortex, however, we observed opposite alterations: a substantial increase in the mRNA of P2ry1. Moreover, we observed an increased mRNA level of the P2ry12 receptor (Figure 6b). These abnormalities in the cortex were confirmed by the protein level for those receptors (Figure 6d).

The analysis of P2Y1 immunostaining in the investigated brain areas showed its co-expression with MAP-positive neurons, whereas the co-expression of P2Y1 with glial marker Iba-1 was not visible. Surprisingly, weak colocalization was observed with the astrocyte marker GFAP, both under control conditions and after treatment with VPA (Figure 6f, Supplementary Figure S2). The specificity of the antibody was confirmed by the absence of immunoreactivity in tissue slices pretreated with the blocking peptide (ALOMONE) (Supplementary Figure S13).

The analysis of P2Y12 co-localisation revealed its predominant presence in the rat hippocampus and prefrontal cortex microglial cells. The immunoreactivity of this receptor was not observed in GFAP-stained astrocytes and MAP-2-stained neuronal processes in each investigated brain area (Figure 6g).

### 3.3. The Impact of Prenatal Exposure to Valproic Acid (VPA) on the Morphology of Astrocytes and Microglia, and the Expression of Pro-Inflammatory Cytokines in the Brain of 52-Day-Old Animals

Our previous data showed that prenatal exposure to VPA resulted in an increase in proinflammatory signalling in the brain of adolescent rats in a region-specific manner [50]. Thus, in this study, we addressed the question of whether the effect of VPA on the changes in purinergic receptor levels may be associated with the activation of glial cells and neuroinflammatory response. Since astrocytes, the most abundant cells within the CNS involved in the regulation of neuroinflammatory response, have been shown to express a variety of purinergic receptor subtypes, we decided to investigate the activity and morphological changes occurring in astrocytes in animals prenatally exposed to VPA.

**Figure 7:**
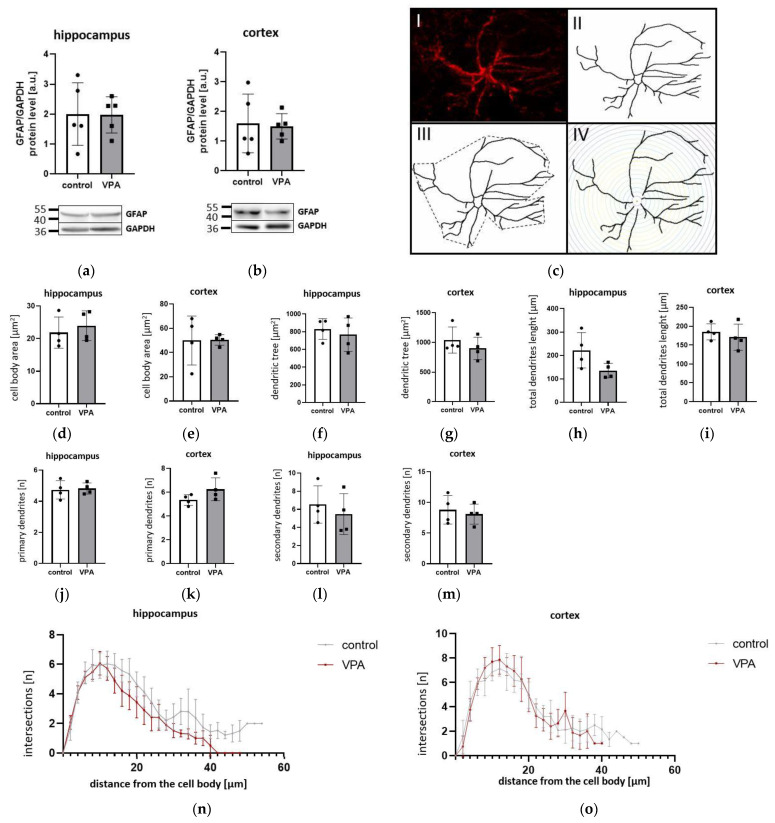
The effect of prenatal treatment with VPA on the morphology of astrocytes in the hippocampus and cortex of 52-day-old rats. The immunoreactivity of GFAP in the hippocampus (**a**) and cortex (**b**) of young adult animals prenatally exposed to VPA treatment was analysed by Western blotting, and Glyceraldehyde-3-phosphate dehydrogenase (GAPDH) was used as a loading control. Representative pictures are shown. Data are presented as the mean value ± SD for 5 independent experiments. The subsequent steps in the morphology analysis of astrocytes are shown (**c**). The image of an GFAP-labelled astrocyte (**I**) was converted to an 8-bit format. Processes were traced by the NeuronJ plugin for Fiji (**II**). The surface area of the binary profile of the cell was marked with a dotted line around the astrocyte (**III**). Digitally applied concentric circles were applied every 2.5 μm, centred on the soma centre (**IV**), and used to count the number of intersections for the Sholl analysis. The surface of the cell body of astrocytes in the hippocampus (**d**) and cortex (**e**) of animals prenatally exposed to VPA was measured. Data are presented as the mean value ± SD for four independent experiments. The dendritic tree of cells in the hippocampus (**f**) and cortex (**g**) of 52-day-old animals prenatally exposed to VPA was analysed as the surface area of cells together with processes. Data are presented as the mean value ± SD for four independent experiments. The total length of processes in the hippocampus (**h**) and cortex (**i**) was measured with the NeuronJ plugin. Data are presented as the mean value ± SD for four independent experiments. The primary dendrites in the hippocampus (**j**) and cortex (**k**), as well as the secondary processes in the hippocampus (**l**) and cortex (**m**) of young adult animals prenatally exposed to VPA treatment, were measured. Data are presented as the mean value ± SD for four independent experiments. Sholl analysis of complexity branching of astrocytes in the hippocampus (**n**) and cortex (**o**) of 52-day-old animals prenatally exposed to VPA treatment was performed with the SNT plugin for Fiji. Data are presented as the mean value ± SD for four independent experiments. Statistical analysis for each data set was determined using Student’s *t*-test.

The characteristic feature of reactive astrocytes is the mRNA and protein overexpression of glial fibrillary acidic protein (GFAP) [54]. We found that the reactive protein levels of this astrocyte marker were not changed in the hippocampus or cortex of the VPA group when compared with the controls (Figure 7a,b). Next, we performed morphometric analysis of GFAP immunolabeled astrocytes in adolescent control and VPA-exposed male rats. Since astrocytes display large heterogeneity in the hippocampus, which appears to be layer-specific [55], we conducted morphological analyses in different layers of the hippocampus. The reconstruction of skeletonized GFAP-positive structures was performed in confocal z-stacks obtained from the CA1 and DG subfields, including the CA1 stratum oriens, stratum lacunosum moleculare, stratum radiatum, DG stratum moleculare, and hilus. The results obtained from each subregion were then averaged. A similar analysis was performed on z-stacks acquired from the prefrontal cortex. We did not observe changes in the cell bodies’ surface area in the investigated brain structures (Figure 7d,e). Using Sholl analysis, it is possible to represent the complexity of the 3D astrocyte structure of the rat brain on a one-dimensional scale (Figure 7c). The analysis revealed that the astrocyte backbone in terms of arbour complexity (Figure 7f,g), total process length (Figure 7h,i), and number of primary (Figure 7j,k) and secondary processes (Figure 7l,m) was not significantly altered in animals prenatally exposed to VPA. We also superimposed spheres of increasing radius (2.5 μm increase in radius per step, Figure 7c) beginning at the centre of the cell body and measured the number of process intersections that each sphere encountered. We found that in both the hippocampus (Figure 7n) and prefrontal cortex (Figure 7o), astrocytes of VPA-exposed animals did not show a significant loss of complexity (the number of intersections at each radius remained unchanged).

**Figure 8:**
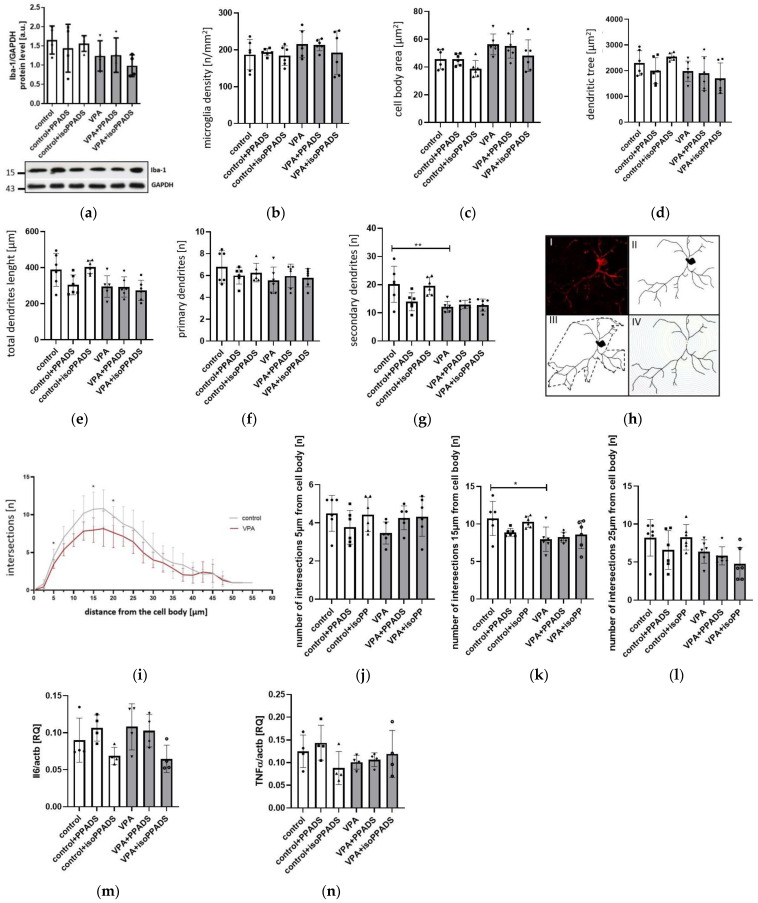
The effect of the inhibition of purinergic signalling on the morphology of microglia and cytokine expression in the hippocampus of 52-day-old rats prenatally treated with VPA. The effect of PPADS/isoPPADS treatment on the immunoreactivity of Iba-1 in the hippocampus (**a**) of young adult animals prenatally exposed to VPA treatment was analysed by Western blotting. Glyceraldehyde-3-phosphate dehydrogenase (GAPDH) was used as a loading control. Representative pictures are shown. Data are presented as the mean value ± SD for four independent experiments. The effect of PPADS/isoPPADS treatment on the density of microglia (**b**) as the number of cells per 1 µm^2^ in the hippocampus of young adult animals prenatally exposed to VPA treatment was measured with the Cell Counter plugin for Fiji. Data are presented as the mean value ± SD for six independent experiments. The effect of PPADS/iso-PPADS treatment on the surface of the cell body of microglia (**c**) and dendritic tree (**d**) in the hippocampus of animals prenatally exposed to VPA was measured. Data are presented as the mean value ± SD for 6 independent experiments. The effect of PPADS/iso-PPADS treatment on the total length of microglial processes (**e**) in the hippocampus of 52-day-old animals prenatally exposed to VPA was measured with the NeuronJ plugin. Data are presented as the mean value ± SD for six independent experiments. The effect of PPADS/iso-PPADS treatment on the number of primary (**f**) and secondary (**g**) dendrites in the hippocampus of young adult animals prenatally exposed to VPA treatment was counted. Data are presented as the mean value ± SD for six independent experiments. * *p* ˂ 0.05, ** *p* < 0.01, vs. corresponding control, as determined using one-way ANOVA followed by the Bonferroni test. The subsequent steps in the morphology analysis of microglia are shown (**h**). The image of the Iba1-labeled microglia cell (**I**) was converted to an 8-bit format. Processes were traced by the NeuronJ plugin for Fiji (**II**). The surface area of the binary profile of the cell was marked with a broken line around the microglia. (**III**) Digitally applied concentric circles were applied every 2.5 μm, centred on the soma centre (**IV**) and were used to count the number of intersections for Sholl analysis. Sholl analysis of complexity branching of microglia (**i**) in the hippocampus of 52-day-old animals prenatally exposed to VPA treatment was performed with the SNT plugin for Fiji. Data are presented as the mean value ± SD for six independent experiments. * *p* ˂ 0.05 vs. corresponding control as determined using Student’s *t*-test. The effect of PPADS/iso-PPADS treatment on the number of intersections 5 μm (**j**), 15 μm (**k**), as well as 25 μm (**l**) from the centre of the cell body, was determined. * *p* ˂ 0.05, vs. corresponding control, as determined using one-way ANOVA followed by the Bonferroni test. The effect of PPADS/iso-PPADS treatment on the mRNA level of Il6 (**m**) and TNFα (**n**) in the hippocampus of young adult animals prenatally exposed to VPA treatment was measured by real-time PCR and normalized to Actb (β-actin). Data are presented as the mean value ± SD for four independent experiments.

To assess the involvement of microglial pathology in the hippocampus of adolescent VPA animals, we first analysed the protein level of Iba-1, but we detected no differences between groups (Figure 8a). Next, we quantified the number and morphological complexity of microglia in the CA1 and DG subfields using confocal microscopy on Iba1-stained sections (Figure 8b). We observed that neither the number of Iba1+ microglial cells per mm2 of tissue in VPA-exposed animals nor their morphology, in terms of a cell body area (Figure 8c), arbour complexity (Figure 8d), and total process length (Figure 8e), displayed significant differences compared to controls. The analysis revealed no differences in the number of primary branches in Iba-1+ cells from VPA-exposed animals compared to control animals (Figure 8f). However, the number of secondary branches significantly decreased in the hippocampi of animals treated with VPA (Figure 8h). This was further evidenced by the decreased arborization of microglia in VPA-exposed rats, especially at 15–20 µm distances from the soma (Figure 8i,k).

Next, we evaluated the mRNA expression levels of interleukin-6 (IL-6) and tumour necrosis factor–α (TNFα), proinflammatory cytokines often linked with microglia activation in ASD. These results demonstrate that in the hippocampus, Il6 and Tnfα mRNA levels were unchanged in VPA-treated animals (Figure 8m,n). We also analysed the involvement of the selective blockade of P2X and overall purinergic P2 receptors with PPADS and isoPPADS, respectively, and observed that the treatment with both drugs had a negligible effect on microglial number, morphology, and activation in control and VPA-treated animals (Figure 8). Additionally, in the hippocampus, these antagonists did not influence the morphological changes of microglial cells evoked by prenatal VPA treatment (Figure 8g,k).

**Figure 9:**
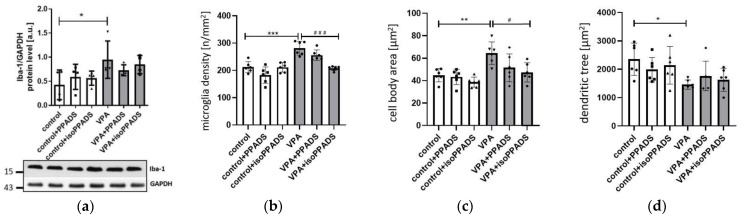

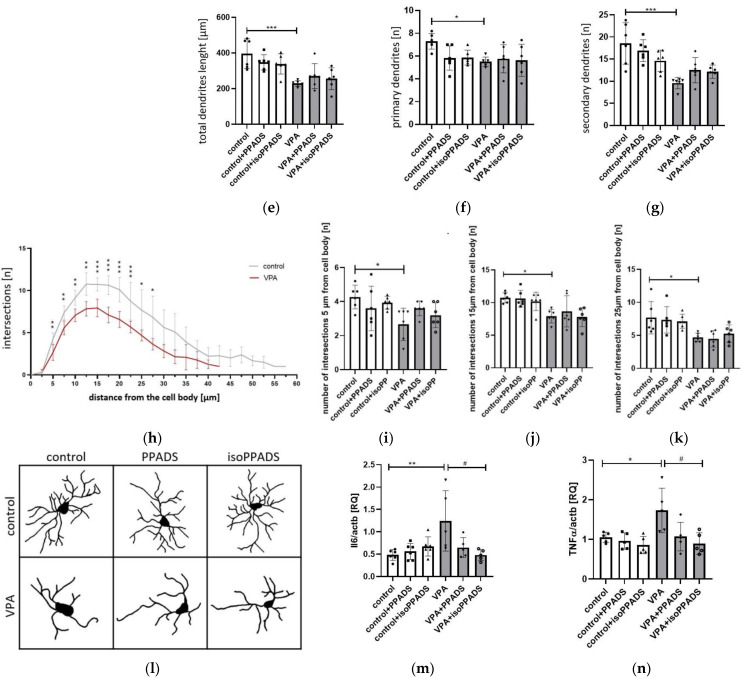
The effect of the inhibition of purinergic signalling on the morphology of microglia and cytokine expression in the cortex of 52-day-old rats prenatally treated with VPA. The effect of PPADS/isoPPADS treatment on the immunoreactivity of Iba-1 in the cortex (**a**) of young adult animals prenatally exposed to VPA treatment was analysed by Western blotting. Glyceraldehyde-3-phosphate dehydrogenase (GAPDH) was used as a loading control. Representative pictures were shown. Data are presented as the mean value ± SD for four independent experiments. The effect of PPADS/iso-PPADS treatment on the density of microglia (**b**) as the number of cells per 1 µm^2^ in the cortex of young adult animals prenatally exposed to VPA treatment was measured with the Cell Counter plugin for Fiji. Data are presented as the mean value ± SD for six independent experiments. * *p* ˂ 0.05, *** *p* < 0.001 vs. corresponding control, # *p* ˂ 0.05, ### *p* < 0.001 vs. VPA, as determined using one-way ANOVA followed by the Bonferroni test. The effect of PPADS/isoPPADS treatment on the surface of the cell body of microglia (**c**) and dendritic tree (**d**) in the cortex of animals prenatally exposed to VPA was measured. Data are presented as the mean value ± SD for six independent experiments. * *p* ˂ 0.05, ** *p* < 0.01 vs. corresponding control, # *p* ˂ 0.05 vs. VPA, as determined using one-way ANOVA followed by the Bonferroni test. The effect of PPADS/isoPPADS treatment on the total length of microglial processes (**e**) in the cortex of 52-day-old animals prenatally exposed to was measured with the NeuronJ plugin. Data are presented as the mean value ± SD for six independent experiments. *** *p* < 0.001 vs. corresponding control, as determined using one-way ANOVA followed by the Bonferroni test. The effect of PPADS/iso-PPADS treatment on the number of primary (**f**) and secondary (**g**) dendrites in the cortex of young adult animals prenatally exposed to VPA treatment was measured. Data are presented as the mean value ± SD for six independent experiments. *** *p* < 0.001, vs. corresponding control as determined using one-way ANOVA followed by the Bonferroni test. Sholl analysis of complexity branching of microglia (**h**) in the cortex of 52-day-old animals prenatally exposed to VPA treatment was performed with the SNT plugin for Fiji. Data are presented as the mean value ± SD for six independent experiments. * *p* ˂ 0.05, ** *p* < 0.01, *** *p* < 0.001 vs. corresponding control, as determined using Student’s *t*-test. The effect of PPADS/iso-PPADS treatment on the number of intersections at 5 μm (**i**), 15 μm (**j**), as well as at 25 μm (**k**) from the centre of the cell body, was determined. * *p* ˂ 0.05 vs. corresponding control, as determined using one-way ANOVA followed by the Bonferroni test. The representative data of microglial cells in the investigated groups are presented (**l**). The effect of PPADS/iso-PPADS treatment on the mRNA level of Il6 (**m**) and TNFα (**n**) in the cortex of young adult animals prenatally exposed to VPA treatment was measured by real-time PCR and normalized to Actb (β-actin). Data are presented as the mean value ± SD for six independent experiments. * *p* ˂ 0.05, # *p* ˂ 0.05 vs. VPA as determined using one-way ANOVA followed by the Bonferroni test.

In contrast to the hippocampus, in the prefrontal cortex, we observed that the protein level for Iba-1 increased in the VPA group (Figure 9a). Moreover, the number of Iba1+ cells per mm^2^ of tissue in VPA animals was significantly higher compared to controls, while treatment with non-selective P2 receptor antagonist isoPPADS resulted in a reduction in the number of microglial cells in VPA-exposed animals to the control level (Figure 9b). Microglial cells in the prefrontal cortex of VPA-treated animals exhibited a typically activated morphology, characterised by a significant increase in the size of microglial cell soma (Figure 9c), a decrease in arbour complexity (Figure 9d), and a decrease in total process length (Figure 9e) compared to the control. We also observed a decrease in the number of primary and secondary branches in Iba-1+ cells (Figure 9f,g) and a reduction in the number of proximal (Figure 9i) and distal (Figure 9i–k) intersections per radius in the microglia of VPA-exposed animals when compared to controls. Interestingly, treatment with the selective P2X purinergic receptor antagonist did not influence the above-mentioned changes in microglial cells morphology, whereas the non-selective P2 antagonist isoPPADS prevented the enlargement of cell bodies of Iba+ cells but had a negligible effect on changes in microglial cell arborisation. However, this compound significantly inhibited the increase in the expression of Il6 and Tnfα induced by prenatal exposure to VPA (Figure 8m,n).

## 4. Discussion

In recent years, it has been shown that purinergic signalling disruption might be involved in the pathomechanisms related to ASDs. The P2X and P2Y receptors are known to control a wide range of molecular processes that are disturbed in autism, such as synaptogenesis and brain development [13], the inflammatory response and microglial activation [56], regulation of intestinal motility and permeability [57,58], chemosensory transduction of taste [59] and hearing [60], and sensitivity to food allergens [61]. It was previously demonstrated that the non-selective blockade of purinergic receptors corrects aberrant behaviour and prevents morphological and biochemical changes in ASD animal models [62,63,64,65]. Following those observations, the present study provides experimental evidence suggesting for the first time that ASD-related disturbances in purinergic signalling might be directly related to changes in the expression of particular subtypes of purinergic receptors occurring in adolescence. Moreover, these alterations might, at least in part, be responsible for microglial activation and neuroinflammatory response induction in the brains of young adult animals.

In this study, we used an environmentally triggered rat models of ASD based on prenatal exposure to valproic acid (VPA), a commonly used antiepileptic drug [66,67]. Several large retrospective studies have shown that VPA intake during pregnancy increases the risk of ASD prevalence almost eightfold when compared to the general population [68,69,70]. Administering VPA to pregnant rodent females at a critical time during gestation results in the development of many structural and behavioural features in the adult offspring that can be observed in ASD patients [50,71]. Because it meets the criteria of construct, face, and predictive validity, the animal model of VPA exposure is considered relevant to a psychiatric condition described in humans and is still one of the most frequently used ASD models [72,73,74]. Numerous studies have documented that the teratogenicity of VPA is connected to the deregulation of epigenetic processes that control the coordinated execution of protein expression involved in neural network maturation [75]. In utero VPA exposure increases histone acetylation and methylation in mouse foetal tissue [76], which can lead to increased synapses and robust facilitation of excitatory synapse maturation [77]. We have previously observed that prenatal exposure to VPA alters the levels of presynaptic and postsynaptic density proteins, as well as synaptic cell adhesion molecules, in adult offspring [50]. Additionally, in that study, VPA exposure induced regional changes in the expression of proinflammatory genes [50]. The current study extends these observations by showing the deregulation of the protein level of the particular subtypes of purinergic receptors in the cortex and hippocampus of the rat offspring as one of the molecular targets of the VPA action. Moreover, our data suggest that the impairment of the purinergic system might partially contribute to the activation of microglia in the cortex of VPA-treated animals.

We observed prominent ASD-related changes in purinergic receptors in early adulthood. These changes in receptor expression may directly or indirectly influence the behavioural or cognitive phenotype of ASD. Overall, alterations in the expression of each purinergic receptor subtype result in the deregulation of cellular Ca^2+^ level, which largely affects social interaction and induces stereotypic behaviour [78]. Moreover, specific receptors belonging to the P2 subfamily regulate unique cellular pathways related to neurotransmission, synaptic plasticity, or the inflammatory response. Therefore, any change in their expression may trigger specific molecular changes related to ASD pathology.

In our study, we found that the purinergic P2X1 receptor was significantly impacted in both the cortex and hippocampus of adult VPA offspring. The level of this receptor was the highest in the adult rat cortex, where P2X1 mediates an appreciable fraction of excitatory synaptic current in ∼85% of layer V neocortical pyramidal neurons [79]. Consistent with an earlier study showing that microglial cells of adult rat brains do not express P2X1 [80], our study demonstrated that this receptor is predominantly expressed in neuronal cells in the prefrontal cortex of adult rats. The down-regulation of this receptor in cortical regions of VPA-treated animals may suggest disturbances in neuronal synaptic transmission in this brain area. Interestingly, in the hippocampus, where the level of P2X1 is significantly increased in VPA offspring, this receptor was shown to stimulate the evoked release of glutamate from purified nerve terminals [81]. Therefore, the overexpression of this receptor in neurons in the hippocampal cornu ammonis or dentate gyrus regions may suggest its indirect role in the elevation of hippocampal LTP. This study greatly extends previous findings, showing that prenatal VPA exposure disrupts neurogenesis and alters the expression levels of multiple proteins associated with glutamatergic and GABAergic neurotransmission in nonhuman primates [82]. 

We also observed a significant increase in the level of P2X3 receptors in the brain cortex of adult VPA offspring. Among other purinergic receptors, the expression of P2X3 is known to rapidly decrease after birth [83], and in our study, we demonstrated that it is relatively low in the brain of weanling and adult rats. Moreover, its expression and function are predominantly attributed to the primary sensory neurons of the spinal cord [84] and are implicated in the modulation of pain sensitivity [85]. Some data suggest that P2X3 receptors can form stable multimers with P2X1 [86] or P2X2 [87] receptors, and antagonists of P2X2/3 and P2X3 have been shown to inhibit glutamate release in the hippocampus, suggesting their role in the modulation of excitatory synaptic transmission [81]. However, little is known about the relevance of the P2X3 receptor in the brain cortex function, and the conditions under which this alteration becomes functionally important remain to be determined. Interestingly, upregulation of P2X3 receptors in a murine model has previously been associated with increased scratching behaviour [88], which is a common problem in autistic patients [89], suggesting that alterations in P2X3 may be connected with sensory hypersensitivity observed in some individuals with ASD.

Previously, the purinergic P2X7 receptor was indicated as a key factor involved in maternal immune-activation-induced autism-like behavioural and biochemical features [90]. Activation of this ligand-gated ion channel, which is sensitive to high extracellular ATP levels, is mainly associated with inflammatory conditions. Its stimulation acts as a cellular signal for inflammasome activation and the post-translational processing of proinflammatory cytokines, including IL-1β, IL-18, TNF-α and IL-6 [91]. Initially, it was believed that the functional expression of the P2X7 receptor was exclusive to oligodendrocytes and microglia in the CNS [92]. However, recent evidence has shown that P2X7 receptors are widely present in neuronal cells [93,94]. Their activation mediates transmembrane ion fluxes that mediate fast excitatory transmission in the hippocampus and primary cerebrocortical neurons. P2X7 is also involved in the release of glutamate, GABA, and ATP from neuronal terminals [11,95,96]. Excessive stimulation of P2X7 receptors leads to increased production of nitric oxide and induces mitochondrial dysfunction in neuronal cells [97]. 

In this study, we demonstrated the presence of P2X7 receptors in the neuronal perikarya of the prefrontal cortex and pyramidal cell layers of the CA and DG in the hippocampus. The level of this receptor was significantly decreased in the hippocampus of VPA offspring. Interestingly, we did not observe significant activation of microglial cells in the hippocampus of VPA offspring, suggesting that the down-regulation of this receptor may also occur in microglia, leading to a lack of activation in these cells. This unexpected effect was also observed in the study of the Naviaux group, where prenatal exposure to poly-I:C resulted in a marked decrease in P2X7 receptor levels [62]. The decrease was normalized after the administration of non-selective purinergic receptor antagonists, such as suramin. The authors of that study suggested that the observed decrease in receptor expression may be a result of chronically elevated levels of ATP and other nucleotides. The idea that the neuronal P2X7 receptor may be overstimulated in ASDs is supported by a study showing that a single dose of a highly selective and brain-permeable P2X7 antagonist, JNJ47965567, alleviated behavioural alterations in the offspring [90]. 

Recent data have suggested the mitochondrial localization of the P2X7 receptor and its involvement in the control of mitochondrial respiration, mitochondrial matrix Ca^2+^ levels, and regulation of the expression pattern of oxidative phosphorylation enzymes [98]. Genetic deletion (P2X7 knockout mice) or subchronic pharmacological inhibition has been shown to increase carbohydrate oxidation [99]. Disturbances in energetic metabolism have been implicated in cell deregulation and the progression of ASDs [100]. The use of the ketogenic diet in ASD patients has also been shown to significantly improve behaviour and intellect [101,102]. Therefore, we hypothesize that the decline in the P2X7 receptor levels after prenatal VPA administration may be involved in the deregulation of energy metabolism in neuronal cells. However, this interesting hypothesis requires further examination using sophisticated quantitative methods.

It has previously been demonstrated that action potentials trigger the axonal release of ATP and glutamate, and the elevated synaptic ATP acts predominantly on astrocytic P2Y1 metabotropic receptors [103]. Surprisingly, our findings showed that under physiological conditions, P2Y1 is predominantly expressed in neuronal cells in the hippocampus or prefrontal cortex. This is in agreement with other researchers showing that the P2Y1 receptor was mainly localized to neuronal cells of the cerebral cortex, cerebellum, hippocampus, caudate nucleus, putamen, globus pallidus, subthalamic nucleus, red nucleus, and midbrain [29,104,105]. Moreover, it has been demonstrated that P2Y1 is expressed in the soma and mossy fibre terminals of the CA3 subfield of the hippocampus [103], and in physiological conditions, it is involved in reducing neurotransmitter release [105]. However, the expression of these receptors significantly increases in astrocytes during reactive astrogliosis, where they play the predominant role in the regulation of single-cell transients and calcium waves [106,107]. P2Y1 expression in astrocytes has been demonstrated to be increased under different pathological conditions, including oxidative stress [108,109] and ischemia [110]. In contrast to those studies, we did not observe a significant increase in P2Y1 levels in the astrocytes in the cortex and hippocampus of VPA animals. What we did notice was a significant elevation of this receptor’s level exclusively in the neuronal cells in the brain cortex with a simultaneous decrease in its level in the hippocampus. It has previously been demonstrated that P2Y1 overactivation in the medial prefrontal cortex significantly alters cognitive abilities in rats, manifesting in a delay in completing tasks and reduced learning abilities [111]. Executive dysfunction and social cognition deficits are often associated with autism in humans [112], therefore it is possible that P2Y1 disturbances might, at least partly, participate in neurocognitive impairment in individuals with ASD.

It is unclear why we did not detect an elevation of P2Y1 expression in astrocytes, but this is partially in agreement with previous studies showing that P2Y1 was significantly upregulated in neurons and microglia, but not in the astrocytes, in status epilepticus [105]. Other studies have also demonstrated the upregulation of P2Y1 in microglial cells following TGF-β treatment [113]. Unlike these reports, we did not observe any increase in the expression of P2Y1 in microglial cells after VPA exposure. However, our study largely extends the previous findings by defining an interesting aspect of P2Y1, which is its overexpression in different cell subtypes depending on the type of insult or pathological state. 

Interestingly, the overexpression of P2Y1 in astrocytes has been predominantly linked to protective effects, such as the prevention of astrocyte damage caused by oxidative stress [108] or the inhibition of neuronal damage induced by IL-6 release [109]. Treatment with the P2Y1 agonist also had beneficial effects in reducing ischemic lesions through an increase in intracellular Ca^2+^ mobilization and enhanced mitochondrial metabolism in astrocytes [110]. In our study, we did not observe significant changes in astrocytic morphology and number, parameters that have been previously shown to be closely associated with the response of astrocytes to different pathological cues, including alterations in the metabolic state of neighbouring cells [114,115]. Therefore, we speculate that in VPA animals, the overexpression of P2Y1 in astrocytes might be somehow blocked, thereby preventing the protective activity of these cells. The lack of proper support from astrocytes may be the cause of neuronal or synaptic alterations, which may lead to the incidence of neuropsychiatric disorders, including autism. This hypothesis is especially interesting in light of the data showing that impaired calcium signalling in astrocytes resulted in atypical social interaction and repetitive behaviour in mice [78]. Astrocyte abnormalities have also been identified post-mortem in the cortex of brains of individuals with autism [35,116,117], and recently, the transplantation of iPSC-derived human ASD astrocytes to the healthy mouse brain was sufficient to induce repetitive behaviour as well as impaired memory and long-term potentiation [118].

Finally, we demonstrated that the expression of the metabotropic P2Y12 is significantly enhanced in the cortex of animals prenatally exposed to VPA. These receptors are generally considered to be a signature of microglia since they are universally and specifically expressed in these cells in all regions of rodent [119] and human brains [120]. The presence of P2Y12 distinguishes CNS resident microglia from peripheral macrophages [22,119]. Our study also showed the specific abundance of this purinergic receptor subtype in the microglial cells in the brains of control and VPA-treated rats. Moreover, we observed that, similar to humans [120], the expression of these receptors is stable across different stages of rat brain development.

The activation of P2Y12 receptors on microglia has been associated with various pathological conditions in the CNS [22,121,122]. Compared to physiological extracellular ATP levels in the brain, high concentrations of this signalling molecule (1 mM) have been shown to induce P2Y12-receptor-dependent prompt chemotaxis of microglia towards the ATP-releasing source [23]. Moreover, stimulation of the P2Y12 receptor in microglia rapidly induces lamellipodia formation, process extension, and morphological conversion [22] into the ramified state. Microglial process convergence towards hyperactivated neurons has been specifically mediated by P2Y12 [31,123], and this receptor also participates in the formation of neuron–microglia junctions, thus regulating mitochondrial function in neurons, neuronal calcium load, and functional connectivity [124]. Therefore, the activation of P2Y12 on microglial cells has been previously considered neuroprotective. Furthermore, robust expression of P2Y12 was demonstrated in the ‘resting’ state of microglia but is dramatically reduced after microglial activation [22]. Activation of microglial TLR-4 receptors by lipopolysaccharide (LPS) has been associated with almost a total loss of P2Y12 receptors [125,126]. Also, gradual and almost complete disappearance of P2Y12 receptors on microglia has been observed in stroke [127]. 

Considering these findings, we expected that P2Y12 overexpression in the cortex of animals prenatally exposed to VPA would result in increased microglial chemotaxis and intensification of microglial process extension. However, while we observed an increase in the number of microglial cells in the cortex of VPA rats mediated by purinergic receptors, the morphology of microglial cells underwent a transition that is characteristic of reactive microgliosis, which has previously been associated with the down-regulation of microglial P2Y12 receptor expression [128], as demonstrated in various neurological disorders [128,129]. However, recent studies have indicated the presence of P2Y12 in activated microglia in post-mortem tissues of patients suffering from major depressive disorder [130] or Alzheimer’s disease [131]. Moreover, it has recently been demonstrated that microglial P2Y12 is a critical mediator of stress-induced neuronal remodelling in the prefrontal cortex and contributes to associated behavioural consequences [132].

Also, in animal models of neuropathic pain, upregulation of the P2Y12 receptor in microglial cells resulted in the activation of the GTP-RhoA/ROCK2 signalling pathway, elevation of phosphorylated p38-mitogen-activated protein kinase (MAPK) levels and upregulation of excitatory synaptic transmission in the dorsal horn [121,133]. Taking into consideration the recent data obtained from the high-resolution single-cell technologies, which reveal the large diversity of microglial cells populations both under homeostatic and pathological conditions [134], it is therefore plausible that epigenetic changes induced by prenatal VPA exposure might result in the formation of P2Y12-positive microglial cells that adopt an activation-specific phenotype. Consistent with this suggestion, it was demonstrated that P2Y12-positive microglia can overexpress pro-inflammatory mediators, such as iNOS, IL-1β, and TNF-α, upon LPS stimulation, and this effect was significantly reduced by P2Y12-inhibitor treatment [135]. In line with that study, we demonstrated that the purinergic receptor antagonist prevented cytokine overexpression in the cortex of animals prenatally exposed to VPA. Previous evidence has shown that aberrant release of cytokines, especially Il-6, alters neural cell adhesion and migration, and also disrupts the balance of excitatory and inhibitory circuits in ASD patients [136]. Our study additionally shows the direct involvement of purinergic receptors in the brain cytokine imbalance, which is closely related to the pathogenesis of autism. Despite the apparent involvement of purinergic signalling in the elevation of cytokine expression in cortical microglial cells, our study revealed that those receptors are not involved in the regulation of morphological changes observed in microglia in the cortex of VPA animals. In light of previous observations showing that microglial process surveillance could also be regulated by other factors, such as β2-adrenergic receptors [137] or changes in extracellular adenosine levels [138], it is possible that the observed changes in microglial morphology could be induced by one of those mechanisms. This is particularly interesting since both β2-adrenergic receptors [139,140] and imbalances in adenosine signalling [141] have previously been shown to be involved in conditions related to ASD. Another noteworthy observation that explains our results is that mutations or epigenetic changes can induce abnormalities in P2Y12 function in platelets, leading to clopidogrel resistance [142,143]. Therefore, prenatal exposure to histone acetylase inhibitor VPA might induce epigenetic changes that result in the expression of dysfunctional P2Y12 receptors. In that case, the overexpression of this receptor in microglial cells might be ineffective in preventing microglial cell de-ramification. This interesting hypothesis appears to be quite probable in light of the data showing valproate-mediated disturbances of haemostasis and platelet function [144] and will be further tested in our future research.

We are aware of the limitations of this study. First, Western blotting analysis of the brain tissue homogenate only allows for the net assessment of changes in particular protein levels without providing information about their alteration in specific cell subtypes and/or cellular compartments, such as pre- or post-synaptic terminals. To partially overcome these limitations, we performed IHC staining on selected purinergic receptors to observe their presence in particular cells within the CNS. Furthermore, we focused on the investigation of the expression of particular receptor subtypes only in a selected stage of CNS development, while purinergic signalling might also be significantly altered in other stages of neuronal development when neurogenesis, neuronal migration, axon growth, and functional axonal maturation occur. Finally, a general limitation of using the VPA rodent model is that the results obtained might not easily translate to the broader human ASD population. Even though prenatal exposure to VPA recapitulates many features characteristic of idiopathic autism, the aetiology of this disorder in the majority of people is not directly connected to prenatal exposure to HDAC inhibitors. Thus, there is a risk that the observed phenomenon may not appear in a certain population of patients. However, the results obtained in this study indicate an urgent need to investigate the changes in purinergic receptors expression and activity in humans suffering from ASD and the relation of these changes to disorder outcomes. This is especially pertinent since a few previous studies showed a certain effectiveness of purinergic receptor inhibition on motor coordination and social impairments in animal ASD models [62,63,65], the genetic Fragile X model [64], as well as in autistic children [145]. Therefore, studying the changes in purinergic neurotransmission in ASD seems to be a promising target for future investigations, especially when various centrally penetrant P2 receptors antagonists are now undergoing clinical trials for the treatment of CNS disorders, such as neuropathic pain, multiple sclerosis, stroke, epilepsy, or Alzheimer’s disease [146,147].

## 5. Conclusions

In summary, our report is the first to show that prenatal exposure to VPA induces significant structure-dependent changes in the expression of purinergic receptors in early adulthood. Moreover, we demonstrated the partial involvement of purinergic signalling imbalance in modulating the brain inflammatory response induced by prenatal VPA exposure. Thus, this study establishes a solid foundation for future investigations into the involvement of purinergic receptor deregulation in ASD, and the development of therapeutic strategies for treatment of this neurodevelopmental disorder.

## Figures and Tables

**Table 1:** A detailed list of used TaqMan Gene Expression Assays for qRT-PCR.

Receptor Gene Symbol	Assay ID	Cat. No.
*Adora1*	Rn00567668_m1	4331182
*Adora2a*	Rn00583935_m1	4331182
*Adora2b*	Rn00567697_m1	4331182
*Adora3*	Rn00563680_m1	4331182
*P2rx1*	Rn00564454_m1	4331182
*P2rx2*	Rn00586491_m1	4331182
*P2rx3*	Rn00579301_m1	4331182
*P2rx4*	Rn00580949_m1	4331182
*P2rx5*	Rn00589966_m1	4331182
*P2rx7*	Rn00570451_m1	4331182
*P2ry1*	Rn00562996_m1	4331182
*P2ry2*	Rn02070661_s1	4331182
*P2ry12*	Rn02133262_s1	4331182
*Il6*	Rn01410330_m1	4331182
*Tnf*	Rn99999017_m1	4331182
*Actb*	Rn00667869_m1	4331182

**Table 2:** A detailed list of used antibodies and employed protocols for Western blotting.

Receptor	Company	Cat. No.	Conditions
A1	Thermo Fischer, Carlsbad, CA, USA	PA1041A	Blocking: 5% milk in TBS-T, 1 h at RT I-ab 1:500 in 5% milk in TBS-T, 2 h at RT II-ab anti-rabbit 1:5000 in 5% milk in TBS-T, 1 h at RT
A2a	Sigma-Aldrich, St. Louis, MO, USA	AB1559P	Blocking: 2% BSA in TBS-T, 1 h at RT I-ab 1:200 in 2% BSA in TBS-T, overnight at 4 °C II-ab anti-rabbit 1:4000 in 5% milk in TBS-T, 1 h at RT
A2b	Sigma-Aldrich, St. Louis, MO, USA	AB1589P	Blocking: 2% BSA in TBS-T, 1 h at RT I-ab 1:250 in 2% BSA in TBS-T, overnight at 4 °C II-ab 1:4000 in 5% milk in TBS-T, 1 h at RT
A3	Sigma-Aldrich, St. Louis, MO, USA	AB1590P	Blocking: 5% BSA in TBS-T, 1 h at RT I-ab 1:500 in 2% BSA in TBS-T, overnight at 4 °C II-ab 1:4000 in 5% milk in TBS-T, 1 h at RT
P2X1	Thermo Fischer, Carlsbad, CA, USA	APR-001	Blocking: 5% BSA in TBS-T, 1 h in RT I-ab 1:500 in 5% BSA in TBS-T, overnight at 4 °C II-ab 1:4000 in 5% milk in TBS-T, 1 h at RT
P2X2	Thermo Fischer, Carlsbad, CA, USA	PA5-119683	Blocking: 5% BSA in PBS, 1 h at RT I-ab 1:500 in 5% BSA in PBS, 2 h at RT II-ab 1:5000 in 5% milk in TBS-T, 1 h at RT
P2X3	Sigma-Aldrich, St. Louis, MO, USA	P0121	Blocking: 2% BSA in TBS-T, 1h at RT I-ab 1:200 in 2% BSA in TBS-T, overnight at 4 °C II-ab 1:4000 in 5% milk in TBS-T, 1 h in RT
P2X4	Alomone Labs, Jerusalem, Israel	APR-024	Blocking: 5% milk in TBS-T, 1 h at RT I-ab 1:400 in 5% BSA in TBS-T, overnight at 4 °C II-ab 1:4000 in 5% milk in TBS-T, 1 h at RT
P2X5	Alomone Labs, Jerusalem, Israel	APR-027	Blocking: 5% milk in TBS-T, 1 h at RT I-ab 1:400 in 5% BSA in TBS-T, overnight at 4 °C II-ab 1:4000 in 5% milk in TBS-T, 1 h at RT
P2X7	Alomone Labs, Jerusalem, Israel	APR-008	Blocking: 5% BSA in TBS-T, 1 h at RT I-ab 1:500 in 5% BSA in TBS-T, overnight at 4 °C II-ab 1:4000 in 5% milk in TBS-T, 1 h at RT
P2Y1	Sigma-Aldrich, St. Louis, MO, USA	P-6487	Blocking: 5% milk in TBS-T, 1 h at RT I-ab 1:100 in TBS-T, overnight at 4 °C II-ab 1:4000 in 5% milk in TBS-T, 1 h in RT
P2Y2	Alomone Labs, Jerusalem, Israel	APR-010	Blocking: 5% milk in TBS-T, 1 h at RT I-ab 1:100 in 2% BSA, overnight at 4 °C II-ab 1:4000 in 5% milk in TBS-T, 1 h at RT
P2Y12	Thermo Fischer, Carlsbad, CA, USA	702516	Blocking: 5% BSA in TBS-T, 1 h at RT I-ab 1:1000 in 5% milk in TBS-T, overnight at 4 °C II-ab 1:1000 in 5% milk in TBS-T, 1 h at RT
GFAP	Abcam, Cambridge, UK	ab53554	Blocking: 5% milk in TBS-T, 1 h at RT I-ab 1:750 in TBS-T, overnight at 4 °C II-ab 1:4000 in 5% milk in TBS-T, 1 h at RT
Iba-1	Cell Signaling, USA	17198	Blocking: 5% milk in TBS-T, 1 h at RT I-ab 1:1000 in TBS-T, overnight at 4 °C II-ab 1:4000 in 5% milk in TBS-T, 1 h at RT
HRP-GAPDH	Proteintech, Rosemont, IL, USA	HRP-60004	Blocking: 5% milk in TBS-T, 1 h at RT I-ab 1:50,000 in 5% milk in TBS-T, 1 h at 4 °C
Goat anti-rabbit	Proteintech, Rosemont, IL, USA	SA00001-2	1:4000 or 1:1000 in 5% milk in TBS-T, 1 h at RT

**Table 3:** Detailed list of used antibodies and employed protocols for immunohistochemistry.

Receptor	Company	Cat. No.	Conditions
P2X1	Alomone Labs, Jerusalem, Israel	APR-022-AG	I-ab 1:50 in PBS containing 1% BSA, 0.3% Triton X; incubation for 1 h at RT, and overnight at 4 °C
P2X7	Alomone Labs, Jerusalem, Israel	APR-004	I-ab 1:100 in PBS containing 1% BSA, 0.3% Triton X; incubation for 1 h at RT, and overnight at 4 °C
P2Y1	Alomone Labs, Jerusalem, Israel	APR-021-AG	I-ab 1:50 in PBS containing 1% BSA, 0.3% Triton X; incubation for 1 h at RT, and overnight at 4 °C
P2Y12	Thermo Fischer, Carlsbad, CA, USA	702516	I-ab 1:200 in PBS containing 1% BSA, 0.3% Triton X; incubation for 1 h at RT, and overnight at 4 °C
MAP-2	Invitrogen, Carlsbad, CA, USA	AP18	I-ab 1:1000 in PBS containing 1% BSA, 0.3% Triton X; incubation for 1 h at RT, and overnight at 4 °C
GFAP	Abcam, Cambridge, UK	ab53554	I-ab 1:500 in PBS containing 1% BSA, 0.3% Triton X; incubation for 1 h at RT, and overnight at 4 °C
Iba-1	Abcam, Cambridge, UK	ab5076	I-ab 1:500 in PBS containing 1% BSA, 0.3% Triton X; incubation for 1 h at RT, and overnight at 4 °C

## Data Availability

Not applicable.

## Supplementary Materials

The following supporting information can be downloaded at: https://www.mdpi.com/2076-3425/13/7/1088/s1. The impact of prenatal exposure to VPA on P2X1 receptor level in the neurons (Figure S1), astrocytes (Figure S2), and microglia (Figure S3) of the hippocampus and the prefrontal cortex at 52 PND; the impact of prenatal exposure to VPA on P2X7 receptor level in the neurons (Figure S4), astrocytes (Figure S5), and microglia (Figure S6) of the hippocampus and the prefrontal cortex at 52 PND; the impact of prenatal exposure to VPA on P2Y1 receptor level in the neurons (Figure S7), astrocytes (Figure S8), and microglia (Figure S9) of the hippocampus and the prefrontal cortex at 52 PND; the impact of prenatal exposure to VPA on P2Y12 receptor level in the neurons (Figure S10), astrocytes (Figure S11), and microglia (Figure S12) of the hippocampus and the prefrontal cortex at 52 PND; antibody specificity (Figure S13).

## References

[1] Burnstock G., Fredholm B.B., North R.A., Verkhratsky A. (2010). The Birth and Postnatal Development of Purinergic Signalling. Acta Physiol..

[2] Mut-Arbona P., Sperlágh B. (2023). P2 Receptor-Mediated Signaling in the Physiological and Pathological Brain: From Development to Aging and Disease. Neuropharmacology.

[3] Agostinho P., Madeira D., Dias L., Simões A.P., Cunha R.A., Canas P.M. (2020). Purinergic Signaling Orchestrating Neuron-Glia Communication. Pharm. Res..

[4] Mahmood A., Iqbal J. (2022). Purinergic Receptors Modulators: An Emerging Pharmacological Tool for Disease Management. Med. Res. Rev..

[5] Zimmermann H. (1994). Signalling via ATP in the Nervous System. Trends Neurosci..

[6] Wang X., Dong Y.-T., Hu X.-M., Zhang J.-Z., Shi N.-R., Zuo Y.-Q., Wang X. (2023). The Circadian Regulation of Extracellular ATP. Purinergic Signal..

[7] Dosch M., Gerber J., Jebbawi F., Beldi G. (2018). Mechanisms of ATP Release by Inflammatory Cells. Int. J. Mol. Sci..

[8] Bodin P., Burnstock G. (2001). Purinergic Signalling: ATP Release. Neurochem. Res..

[9] Taruno A. (2018). ATP Release Channels. Int. J. Mol. Sci..

[10] Baroja-Mazo A., Barberà-Cremades M., Pelegrín P. (2013). The Participation of Plasma Membrane Hemichannels to Purinergic Signaling. Biochim. Biophys. Acta BBA Biomembr..

[11] Wilkaniec A., Gąssowska M., Czapski G.A., Cieślik M., Sulkowski G., Adamczyk A. (2017). P2X7 Receptor-Pannexin 1 Interaction Mediates Extracellular Alpha-Synuclein-Induced ATP Release in Neuroblastoma SH-SY5Y Cells. Purinergic Signal..

[12] Horvat A., Stanojević I., Drakulić D., Veličković N., Petrović S., Milošević M. (2010). Effect of Acute Stress on NTPDase and 5′-nucleotidase Activities in Brain Synaptosomes in Different Stages of Development. Int. J. Dev. Neurosci..

[13] Abbracchio M.P., Burnstock G., Verkhratsky A., Zimmermann H. (2009). Purinergic Signalling in the Nervous System: An Overview. Trends Neurosci..

[14] Burnstock G. (2007). Physiology and Pathophysiology of Purinergic Neurotransmission. Physiol. Rev..

[15] Burnstock G., Dale N. (2015). Purinergic Signalling during Development and Ageing. Purinergic Signal..

[16] Mishra S.K., Braun N., Shukla V., Füllgrabe M., Schomerus C., Korf H.-W., Gachet C., Ikehara Y., Sévigny J., Robson S.C. (2006). Extracellular Nucleotide Signaling in Adult Neural Stem Cells: Synergism with Growth Factor-Mediated Cellular Proliferation. Development.

[17] Masino S.A., Kawamura M., Cote J.L., Williams R.B., Ruskin D.N. (2013). Adenosine and Autism: A Spectrum of Opportunities. Neuropharmacology.

[18] Merighi S., Poloni T.E., Terrazzan A., Moretti E., Gessi S., Ferrari D. (2021). Alzheimer and Purinergic Signaling: Just a Matter of Inflammation?. Cells.

[19] Fields R.D., Burnstock G. (2006). Purinergic Signalling in Neuron–Glia Interactions. Nat. Rev. Neurosci..

[20] Stevens B., Fields R.D. (2000). Response of Schwann Cells to Action Potentials in Development. Science.

[21] Stevens B., Porta S., Haak L.L., Gallo V., Fields R.D. (2002). Adenosine. Neuron.

[22] Haynes S.E., Hollopeter G., Yang G., Kurpius D., Dailey M.E., Gan W.-B., Julius D. (2006). The P2Y12 Receptor Regulates Microglial Activation by Extracellular Nucleotides. Nat. Neurosci..

[23] Davalos D., Grutzendler J., Yang G., Kim J.V., Zuo Y., Jung S., Littman D.R., Dustin M.L., Gan W.-B. (2005). ATP Mediates Rapid Microglial Response to Local Brain Injury in Vivo. Nat. Neurosci..

[24] Hide I., Tanaka M., Inoue A., Nakajima K., Kohsaka S., Inoue K., Nakata Y. (2002). Extracellular ATP Triggers Tumor Necrosis Factor-α Release from Rat Microglia. J. Neurochem..

[25] Vainchtein I.D., Molofsky A.V. (2020). Astrocytes and Microglia: In Sickness and in Health. Trends Neurosci..

[26] Guthrie P.B., Knappenberger J., Segal M., Bennett M.V.L., Charles A.C., Kater S.B. (1999). ATP Released from Astrocytes Mediates Glial Calcium Waves. J. Neurosci..

[27] Shinozaki Y., Shibata K., Yoshida K., Shigetomi E., Gachet C., Ikenaka K., Tanaka K.F., Koizumi S. (2017). Transformation of Astrocytes to a Neuroprotective Phenotype by Microglia via P2Y1 Receptor Downregulation. Cell Rep..

[28] Ribeiro D.E., Petiz L.L., Glaser T., Oliveira-Giacomelli Á., Andrejew R., Saab F.D., da Silva Milanis M., Campos H.C., Sampaio V.F., La Banca S. (2023). Purinergic Signaling in Cognitive Impairment and Neuropsychiatric Symptoms of Alzheimer’s Disease. Neuropharmacology.

[29] Puerto A.D., Wandosell F., Garrido J.J. (2013). Neuronal and Glial Purinergic Receptors Functions in Neuron Development and Brain Disease. Front. Cell Neurosci..

[30] Koizumi S. (2022). Glial Purinergic Signals and Psychiatric Disorders. Front. Cell Neurosci..

[31] Zimmermann F.F., Gaspary K.V., Siebel A.M., Leite C.E., Kist L.W., Bogo M.R., Bonan C.D. (2017). Analysis of Extracellular Nucleotide Metabolism in Adult Zebrafish After Embryological Exposure to Valproic Acid. Mol. Neurobiol..

[32] Petrelli F., Pucci L., Bezzi P. (2016). Astrocytes and Microglia and Their Potential Link with Autism Spectrum Disorders. Front. Cell Neurosci..

[33] Norden D.M., Trojanowski P.J., Villanueva E., Navarro E., Godbout J.P. (2016). Sequential Activation of Microglia and Astrocyte Cytokine Expression Precedes Increased Iba-1 or GFAP Immunoreactivity Following Systemic Immune Challenge. Glia.

[34] Kinoshita S., Koyama R. (2021). Pro- and Anti-Epileptic Roles of Microglia. Neural Regen. Res..

[35] Edmonson C., Ziats M.N., Rennert O.M. (2014). Altered Glial Marker Expression in Autistic Post-Mortem Prefrontal Cortex and Cerebellum. Mol. Autism..

[36] Dai S., Lin J., Hou Y., Luo X., Shen Y., Ou J. (2023). Purine Signaling Pathway Dysfunction in Autism Spectrum Disorders: Evidence from Multiple Omics Data. Front. Mol. Neurosci..

[37] Nicolini C., Fahnestock M. (2018). The Valproic Acid-Induced Rodent Model of Autism. Exp. Neurol..

[38] Young K., Morrison H. (2018). Quantifying Microglia Morphology from Photomicrographs of Immunohistochemistry Prepared Tissue Using ImageJ. J. Vis. Exp..

[39] Sanagi T., Sasaki T., Nakagaki K., Minamimoto T., Kohsaka S., Ichinohe N. (2019). Segmented Iba1-Positive Processes of Microglia in Autism Model Marmosets. Front. Cell Neurosci..

[40] Schindelin J., Arganda-Carreras I., Frise E., Kaynig V., Longair M., Pietzsch T., Preibisch S., Rueden C., Saalfeld S., Schmid B. (2012). Fiji: An Open-Source Platform for Biological-Image Analysis. Nat. Methods.

[41] SHOLL D.A. (1953). Dendritic Organization in the Neurons of the Visual and Motor Cortices of the Cat. J. Anat..

[42] Kerkhofs A., Canas P.M., Timmerman A.J., Heistek T.S., Real J.I., Xavier C., Cunha R.A., Mansvelder H.D., Ferreira S.G. (2018). Adenosine A2A Receptors Control Glutamatergic Synaptic Plasticity in Fast Spiking Interneurons of the Prefrontal Cortex. Front. Pharm..

[43] Gundelfinger E.D., Frischknecht R., Choquet D., Heine M. (2010). Converting Juvenile into Adult Plasticity: A Role for the Brain’s Extracellular Matrix. Eur. J. Neurosci..

[44] Guo W., Xu X., Gao X., Burnstock G., He C., Xiang Z. (2008). Expression of P2X5 Receptors in the Mouse CNS. Neuroscience.

[45] Bo X., Jiang L.-H., Wilson H.L., Kim M., Burnstock G., Surprenant A., North R.A. (2003). Pharmacological and Biophysical Properties of the Human P2X_5_ Receptor. Mol. Pharm..

[46] Kaczmarek-Hájek K., Lörinczi É., Hausmann R., Nicke A. (2012). Molecular and Functional Properties of P2X Receptors—Recent Progress and Persisting Challenges. Purinergic Signal..

[47] Guzman S.J., Gerevich Z. (2016). P2Y Receptors in Synaptic Transmission and Plasticity: Therapeutic Potential in Cognitive Dysfunction. Neural Plast..

[48] Weisman G.A., Woods L.T., Erb L., Seye C.I. (2012). P2Y Receptors in the Mammalian Nervous System: Pharmacology, Ligands and Therapeutic Potential. CNS Neurol. Disord. Drug Targets.

[49] Saitow F., Murakoshi T., Suzuki H., Konishi S. (2005). Metabotropic P2Y Purinoceptor-Mediated Presynaptic and Postsynaptic Enhancement of Cerebellar GABAergic Transmission. J. Neurosci..

[50] Gąssowska-Dobrowolska M., Cieślik M., Czapski G.A., Jęśko H., Frontczak-Baniewicz M., Gewartowska M., Dominiak A., Polowy R., Filipkowski R.K., Babiec L. (2020). Prenatal Exposure to Valproic Acid Affects Microglia and Synaptic Ultrastructure in a Brain-Region-Specific Manner in Young-Adult Male Rats: Relevance to Autism Spectrum Disorders. Int. J. Mol. Sci..

[51] Babiec L., Wilkaniec A., Adamczyk A. (2022). Prenatal Exposure to Valproic Acid Induces Alterations in the Expression and Activity of Purinergic Receptors in the Embryonic Rat Brain. Folia Neuropathol..

[52] Szabó D., Tod P., Gölöncsér F., Román V., Lendvai B., Otrokocsi L., Sperlágh B. (2022). Maternal P2X7 Receptor Inhibition Prevents Autism-like Phenotype in Male Mouse Offspring through the NLRP3-IL-1β Pathway. Brain Behav. Immun..

[53] Hausmann R., Schmalzing G. (2012). P2X1 and P2X2 Receptors in the Central Nervous System as Possible Drug Targets. CNS Neurol. Disord. Drug Targets.

[54] Sofroniew M.V. (2015). Astrogliosis. Cold Spring Harb. Perspect. Biol..

[55] Viana J.F., Machado J.L., Abreu D.S., Veiga A., Barsanti S., Tavares G., Martins M., Sardinha V.M., Guerra-Gomes S., Domingos C. (2023). Astrocyte Structural Heterogeneity in the Mouse Hippocampus. Glia.

[56] Pelegrin P. (2008). Targeting Interleukin-1 Signaling in Chronic Inflammation: Focus on P2X(7) Receptor and Pannexin-1. Drug News Perspect..

[57] Gallego D., Vanden Berghe P., Farré R., Tack J., Jiménez M. (2007). P2Y_1_ Receptors Mediate Inhibitory Neuromuscular Transmission and Enteric Neuronal Activation in Small Intestine. Neurogastroenterol. Motil..

[58] Matos J.E., Sorensen M.V., Geyti C.S., Robaye B., Boeynaems J.M., Leipziger J. (2007). Distal Colonic Na+ Absorption Inhibited by Luminal P2Y2 Receptors. Pflug. Arch..

[59] Surprenant A., North R.A. (2009). Signaling at Purinergic P2X Receptors. Annu. Rev. Physiol..

[60] Housley G.D., Jagger D.J., Greenwood D., Raybould N.P., Salih S.G., Järlebark L.E., Vlajkovic S.M., Kanjhan R., Nikolic P., Muñoz D.J.M. (2002). Purinergic Regulation of Sound Transduction and Auditory Neurotransmission. Audiol. Neurotol..

[61] Leng Y., Yamamoto T., Kadowaki M. (2008). Alteration of Cholinergic, Purinergic and Sensory Neurotransmission in the Mouse Colon of Food Allergy Model. Neurosci. Lett..

[62] Naviaux R.K., Zolkipli Z., Wang L., Nakayama T., Naviaux J.C., Le T.P., Schuchbauer M.A., Rogac M., Tang Q., Dugan L.L. (2013). Antipurinergic Therapy Corrects the Autism-like Features in the Poly(IC) Mouse Model. PLoS ONE.

[63] Naviaux J.C., Schuchbauer M.A., Li K., Wang L., Risbrough V.B., Powell S.B., Naviaux R.K. (2014). Reversal of Autism-like Behaviors and Metabolism in Adult Mice with Single-Dose Antipurinergic Therapy. Transl. Psychiatry.

[64] Naviaux J.C., Wang L., Li K., Bright A., Alaynick W.A., Williams K.R., Powell S.B., Naviaux R.K. (2015). Antipurinergic Therapy Corrects the Autism-like Features in the Fragile X (Fmr1 Knockout) Mouse Model. Mol. Autism..

[65] Hirsch M.M., Deckmann I., Santos-Terra J., Staevie G.Z., Fontes-Dutra M., Carello-Collar G., Körbes-Rockenbach M., Brum Schwingel G., Bauer-Negrini G., Rabelo B. (2020). Effects of Single-Dose Antipurinergic Therapy on Behavioral and Molecular Alterations in the Valproic Acid-Induced Animal Model of Autism. Neuropharmacology.

[66] Felix-Ortiz A.C., Febo M. (2012). Gestational Valproate Alters BOLD Activation in Response to Complex Social and Primary Sensory Stimuli. PLoS ONE.

[67] Rodier P.M., Ingram J.L., Tisdale B., Nelson S., Romano J. (1996). Embryological Origin for Autism: Developmental Anomalies of the Cranial Nerve Motor Nuclei. J. Comp. Neurol..

[68] Moore S.J. (2000). A Clinical Study of 57 Children with Fetal Anticonvulsant Syndromes. J. Med. Genet..

[69] Rasalam A., Hailey H., Williams J., Moore S., Turnpenny P., Lloyd D., Dean J. (2005). Characteristics of Fetal Anticonvulsant Syndrome Associated Autistic Disorder. Dev. Med. Child. Neurol..

[70] Dean J.C.S. (2002). Long Term Health and Neurodevelopment in Children Exposed to Antiepileptic Drugs before Birth. J. Med. Genet..

[71] Roullet F.I., Lai J.K.Y., Foster J.A. (2013). In Utero Exposure to Valproic Acid and Autism—A Current Review of Clinical and Animal Studies. Neurotoxicol. Teratol..

[72] Chaliha D., Albrecht M., Vaccarezza M., Takechi R., Lam V., Al-Salami H., Mamo J. (2020). A Systematic Review of the Valproic-Acid-Induced Rodent Model of Autism. Dev. Neurosci..

[73] Schneider T., Turczak J., Przewłocki R. (2006). Environmental Enrichment Reverses Behavioral Alterations in Rats Prenatally Exposed to Valproic Acid: Issues for a Therapeutic Approach in Autism. Neuropsychopharmacology.

[74] Woo C.C., Leon M. (2013). Environmental Enrichment as an Effective Treatment for Autism: A Randomized Controlled Trial. Behav. Neurosci..

[75] Fukuchi M., Nii T., Ishimaru N., Minamino A., Hara D., Takasaki I., Tabuchi A., Tsuda M. (2009). Valproic Acid Induces Up- or down-Regulation of Gene Expression Responsible for the Neuronal Excitation and Inhibition in Rat Cortical Neurons through Its Epigenetic Actions. Neurosci. Res..

[76] Tung E.W.Y., Winn L.M. (2010). Epigenetic Modifications in Valproic Acid-Induced Teratogenesis. Toxicol. Appl. Pharm..

[77] Akhtar M.W., Raingo J., Nelson E.D., Montgomery R.L., Olson E.N., Kavalali E.T., Monteggia L.M. (2009). Histone Deacetylases 1 and 2 Form a Developmental Switch That Controls Excitatory Synapse Maturation and Function. J. Neurosci..

[78] Wang Q., Kong Y., Wu D.-Y., Liu J.-H., Jie W., You Q.-L., Huang L., Hu J., Chu H.-D., Gao F. (2021). Impaired Calcium Signaling in Astrocytes Modulates Autism Spectrum Disorder-like Behaviors in Mice. Nat. Commun..

[79] Pankratov Y., Lalo U., Krishtal O., Verkhratsky A. (2003). P2X Receptor-Mediated Excitatory Synaptic Currents in Somatosensory Cortex. Mol. Cell. Neurosci..

[80] Xiang Z., Burnstock G. (2005). Expression of Expression of P2X Receptors on Rat Microglial Cells during Early Development. Glia.

[81] Rodrigues R.J., Almeida T., Richardson P.J., Oliveira C.R., Cunha R.A. (2005). Dual Presynaptic Control by ATP of Glutamate Release via Facilitatory P2X1, P2X2/3, and P2X3 and Inhibitory P2Y1, P2Y2, and/or P2Y4 Receptors in the Rat Hippocampus. J. Neurosci..

[82] Zhao H., Wang Q., Yan T., Zhang Y., Xu H., Yu H., Tu Z., Guo X., Jiang Y., Li X. (2019). Maternal Valproic Acid Exposure Leads to Neurogenesis Defects and Autism-like Behaviors in Non-Human Primates. Transl. Psychiatry.

[83] Cheung K.K., Chan W.Y., Burnstock G. (2005). Expression of P2X Purinoceptors during Rat Brain Development and Their Inhibitory Role on Motor Axon Outgrowth in Neural Tube Explant Cultures. Neuroscience.

[84] Vulchanova L., Riedl M.S., Shuster S.J., Stone L.S., Hargreaves K.M., Buell G., Surprenant A., North R.A., Elde R. (1998). P2X_3_ Is Expressed by DRG Neurons That Terminate in Inner Lamina II. Eur. J. Neurosci..

[85] Fabbretti E. (2013). ATP P2X3 Receptors and Neuronal Sensitization. Front. Cell Neurosci..

[86] Nicke A. (1998). P2X1 and P2X3 Receptors Form Stable Trimers: A Novel Structural Motif of Ligand-Gated Ion Channels. EMBO J..

[87] Liu M., King B.F., Dunn P.M., Rong W., Townsend-Nicholson A., Burnstock G. (2001). Coexpression of P2X(3) and P2X(2) Receptor Subunits in Varying Amounts Generates Heterogeneous Populations of P2X Receptors That Evoke a Spectrum of Agonist Responses Comparable to That Seen in Sensory Neurons. J. Pharm. Exp..

[88] Shiratori-Hayashi M., Hasegawa A., Toyonaga H., Andoh T., Nakahara T., Kido-Nakahara M., Furue M., Kuraishi Y., Inoue K., Dong X. (2019). Role of P2X3 Receptors in Scratching Behavior in Mouse Models. J. Allergy Clin. Immunol..

[89] Matson J.L., Rivet T.T. (2007). A Validity Study of the Autism Spectrum Disorders—Behavior Problems for Adults (ASD-BPA) Scale. J. Dev. Phys. Disabil..

[90] Horváth G., Otrokocsi L., Bekő K., Baranyi M., Kittel Á., Antonio Fritz-Ruenes P., Sperlágh B. (2019). P2X7 Receptors Drive Poly(I:C) Induced Autism-like Behavior in Mice. J. Neurosci..

[91] Lister M.F., Sharkey J., Sawatzky D.A., Hodgkiss J.P., Davidson D.J., Rossi A.G., Finlayson K. (2007). The Role of the Purinergic P2X7 Receptor in Inflammation. J. Inflamm..

[92] Kaczmarek-Hajek K., Zhang J., Kopp R., Grosche A., Rissiek B., Saul A., Bruzzone S., Engel T., Jooss T., Krautloher A. (2018). Re-Evaluation of Neuronal P2X7 Expression Using Novel Mouse Models and a P2X7-Specific Nanobody. eLife.

[93] Yu Y., Ugawa S., Ueda T., Ishida Y., Inoue K., Kyaw Nyunt A., Umemura A., Mase M., Yamada K., Shimada S. (2008). Cellular Localization of P2X7 Receptor MRNA in the Rat Brain. Brain Res..

[94] Deuchars S.A., Atkinson L., Brooke R.E., Musa H., Milligan C.J., Batten T.F., Buckley N.J., Parson S.H., Deuchars J. (2001). Neuronal P2X7 Receptors Are Targeted to Presynaptic Terminals in the Central and Peripheral Nervous Systems. J. Neurosci..

[95] Sperlágh B., Köfalvi A., Deuchars J., Atkinson L., Milligan C.J., Buckley N.J., Vizi E.S. (2002). Involvement of P2X7 Receptors in the Regulation of Neurotransmitter Release in the Rat Hippocampus. J. Neurochem..

[96] Wirkner K., Köfalvi A., Fischer W., Günther A., Franke H., Gröger-Arndt H., Nörenberg W., Madarász E., Vizi E.S., Schneider D. (2005). Supersensitivity of P2X Receptors in Cerebrocortical Cell Cultures after in Vitro Ischemia. J. Neurochem..

[97] Wilkaniec A., Cieślik M., Murawska E., Babiec L., Gąssowska-Dobrowolska M., Pałasz E., Jęśko H., Adamczyk A. (2020). P2X7 Receptor Is Involved in Mitochondrial Dysfunction Induced by Extracellular Alpha Synuclein in Neuroblastoma SH-SY5Y Cells. Int. J. Mol. Sci..

[98] Sarti A.C., Vultaggio-Poma V., Falzoni S., Missiroli S., Giuliani A.L., Boldrini P., Bonora M., Faita F., Di Lascio N., Kusmic C. (2021). Mitochondrial P2X7 Receptor Localization Modulates Energy Metabolism Enhancing Physical Performance. Function.

[99] Coccurello R., Volonté C. (2020). P2X7 Receptor in the Management of Energy Homeostasis: Implications for Obesity, Dyslipidemia, and Insulin Resistance. Front. Endocrinol..

[100] Vallée A., Vallée J.-N. (2018). Warburg Effect Hypothesis in Autism Spectrum Disorders. Mol. Brain.

[101] Żarnowska I., Chrapko B., Gwizda G., Nocuń A., Mitosek-Szewczyk K., Gasior M. (2018). Therapeutic Use of Carbohydrate-Restricted Diets in an Autistic Child; a Case Report of Clinical and 18FDG PET Findings. Metab. Brain Dis..

[102] Evangeliou A., Vlachonikolis I., Mihailidou H., Spilioti M., Skarpalezou A., Makaronas N., Prokopiou A., Christodoulou P., Liapi-Adamidou G., Helidonis E. (2003). Application of a Ketogenic Diet in Children with Autistic Behavior: Pilot Study. J. Child. Neurol..

[103] Hamilton N., Vayro S., Kirchhoff F., Verkhratsky A., Robbins J., Gorecki D.C., Butt A.M. (2008). Mechanisms of ATP- and Glutamate-Mediated Calcium Signaling in White Matter Astrocytes. Glia.

[104] Moore D., Chambers J., Waldvogel H., Faull R., Emson P. (2000). Regional and Cellular Distribution of the P2Y1 Purinergic Receptor in the Human Brain: Striking Neuronal Localisation. J. Comp. Neurol..

[105] Alves M., De Diego Garcia L., Conte G., Jimenez-Mateos E.M., D’Orsi B., Sanz-Rodriguez A., Prehn J.H.M., Henshall D.C., Engel T. (2019). Context-Specific Switch from Anti- to Pro-Epileptogenic Function of the P2Y_1_ Receptor in Experimental Epilepsy. J. Neurosci..

[106] Delekate A., Füchtemeier M., Schumacher T., Ulbrich C., Foddis M., Petzold G.C. (2014). Metabotropic P2Y1 Receptor Signalling Mediates Astrocytic Hyperactivity in Vivo in an Alzheimer’s Disease Mouse Model. Nat. Commun..

[107] Kuboyama K., Harada H., Tozaki-Saitoh H., Tsuda M., Ushijima K., Inoue K. (2011). Astrocytic P2Y_1_ Receptor Is Involved in the Regulation of Cytokine/Chemokine Transcription and Cerebral Damage in a Rat Model of Cerebral Ischemia. J. Cereb. Blood Flow Metab..

[108] Shinozaki Y., Koizumi S., Ohno Y., Nagao T., Inoue K. (2006). Extracellular ATP Counteracts the ERK1/2-Mediated Death-Promoting Signaling Cascades in Astrocytes. Glia.

[109] Fujita T., Tozaki-Saitoh H., Inoue K. (2009). P2Y_1_ Receptor Signaling Enhances Neuroprotection by Astrocytes against Oxidative Stress via IL-6 Release in Hippocampal Cultures. Glia.

[110] Zheng W., Watts L.T., Holstein D.M., Wewer J., Lechleiter J.D. (2013). P2Y_1_ R-Initiated, IP_3_ R-Dependent Stimulation of Astrocyte Mitochondrial Metabolism Reduces and Partially Reverses Ischemic Neuronal Damage in Mouse. J. Cereb. Blood Flow Metab..

[111] Koch H., Bespalov A., Drescher K., Franke H., Krügel U. (2015). Impaired Cognition after Stimulation of P2Y1 Receptors in the Rat Medial Prefrontal Cortex. Neuropsychopharmacology.

[112] Hajri M., Abbes Z., Yahia H.B., Jelili S., Halayem S., Mrabet A., Bouden A. (2022). Cognitive Deficits in Children with Autism Spectrum Disorders: Toward an Integrative Approach Combining Social and Non-Social Cognition. Front. Psychiatry.

[113] De Simone R., Niturad C.E., De Nuccio C., Ajmone-Cat M.A., Visentin S., Minghetti L. (2010). TGF-β and LPS Modulate ADP-Induced Migration of Microglial Cells through P2Y1 and P2Y12 Receptor Expression. J. Neurochem..

[114] Gzielo K., Soltys Z., Rajfur Z., Setkowicz Z.K. (2019). The Impact of the Ketogenic Diet on Glial Cells Morphology. A Quantitative Morphological Analysis. Neuroscience.

[115] Gzielo K., Kielbinski M., Ploszaj J., Janeczko K., Gazdzinski S.P., Setkowicz Z. (2017). Long-Term Consumption of High-Fat Diet in Rats: Effects on Microglial and Astrocytic Morphology and Neuronal Nitric Oxide Synthase Expression. Cell Mol. Neurobiol..

[116] Gupta S., Ellis S.E., Ashar F.N., Moes A., Bader J.S., Zhan J., West A.B., Arking D.E. (2014). Transcriptome Analysis Reveals Dysregulation of Innate Immune Response Genes and Neuronal Activity-Dependent Genes in Autism. Nat. Commun..

[117] Vargas D.L., Nascimbene C., Krishnan C., Zimmerman A.W., Pardo C.A. (2005). Neuroglial Activation and Neuroinflammation in the Brain of Patients with Autism. Ann. Neurol..

[118] Allen M., Huang B.S., Notaras M.J., Lodhi A., Barrio-Alonso E., Lituma P.J., Wolujewicz P., Witztum J., Longo F., Chen M. (2022). Astrocytes Derived from ASD Individuals Alter Behavior and Destabilize Neuronal Activity through Aberrant Ca2+ Signaling. Mol. Psychiatry.

[119] Sasaki Y., Hoshi M., Akazawa C., Nakamura Y., Tsuzuki H., Inoue K., Kohsaka S. (2003). Selective Expression of Gi/o-Coupled ATP Receptor P2Y12 in Microglia in Rat Brain. Glia.

[120] Mildner A., Huang H., Radke J., Stenzel W., Priller J. (2017). P2Y_12_ Receptor Is Expressed on Human Microglia under Physiological Conditions throughout Development and Is Sensitive to Neuroinflammatory Diseases. Glia.

[121] Kobayashi K., Yamanaka H., Fukuoka T., Dai Y., Obata K., Noguchi K. (2008). P2Y_12_ Receptor Upregulation in Activated Microglia Is a Gateway of P38 Signaling and Neuropathic Pain. J. Neurosci..

[122] Illes P., Rubini P., Ulrich H., Zhao Y., Tang Y. (2020). Regulation of Microglial Functions by Purinergic Mechanisms in the Healthy and Diseased CNS. Cells.

[123] Gevi F., Zolla L., Gabriele S., Persico A.M. (2016). Urinary Metabolomics of Young Italian Autistic Children Supports Abnormal Tryptophan and Purine Metabolism. Mol. Autism..

[124] Cserép C., Pósfai B., Lénárt N., Fekete R., László Z.I., Lele Z., Orsolits B., Molnár G., Heindl S., Schwarcz A.D. (2020). Microglia Monitor and Protect Neuronal Function through Specialized Somatic Purinergic Junctions. Science.

[125] Fukumoto Y., Tanaka K.F., Parajuli B., Shibata K., Yoshioka H., Kanemaru K., Gachet C., Ikenaka K., Koizumi S., Kinouchi H. (2019). Neuroprotective Effects of Microglial P2Y_1_ Receptors against Ischemic Neuronal Injury. J. Cereb. Blood Flow Metab..

[126] Banerjee P., Paza E., Perkins E.M., James O.G., Kenkhuis B., Lloyd A.F., Burr K., Story D., Yusuf D., He X. (2020). Generation of Pure Monocultures of Human Microglia-like Cells from Induced Pluripotent Stem Cells. Stem. Cell Res..

[127] Kluge M.G., Abdolhoseini M., Zalewska K., Ong L.K., Johnson S.J., Nilsson M., Walker F.R. (2019). Spatiotemporal Analysis of Impaired Microglia Process Movement at Sites of Secondary Neurodegeneration Post-Stroke. J. Cereb. Blood Flow Metab..

[128] Zrzavy T., Hametner S., Wimmer I., Butovsky O., Weiner H.L., Lassmann H. (2017). Loss of ‘Homeostatic’ Microglia and Patterns of Their Activation in Active Multiple Sclerosis. Brain.

[129] Michailidou I., Naessens D.M.P., Hametner S., Guldenaar W., Kooi E.-J., Geurts J.J.G., Baas F., Lassmann H., Ramaglia V. (2017). Complement C3 on Microglial Clusters in Multiple Sclerosis Occur in Chronic but Not Acute Disease: Implication for Disease Pathogenesis. Glia.

[130] Böttcher C., Fernández-Zapata C., Snijders G.J.L., Schlickeiser S., Sneeboer M.A.M., Kunkel D., De Witte L.D., Priller J. (2020). Single-Cell Mass Cytometry of Microglia in Major Depressive Disorder Reveals a Non-Inflammatory Phenotype with Increased Homeostatic Marker Expression. Transl. Psychiatry.

[131] Walker D.G., Tang T.M., Mendsaikhan A., Tooyama I., Serrano G.E., Sue L.I., Beach T.G., Lue L.-F. (2020). Patterns of Expression of Purinergic Receptor P2RY12, a Putative Marker for Non-Activated Microglia, in Aged and Alzheimer’s Disease Brains. Int. J. Mol. Sci..

[132] Bollinger J.L., Dadosky D.T., Flurer J.K., Rainer I.L., Woodburn S.C., Wohleb E.S. (2022). Microglial P2Y12 Mediates Chronic Stress-Induced Synapse Loss in the Prefrontal Cortex and Associated Behavioral Consequences in Male Mice. bioRxiv.

[133] Yu T., Zhang X., Shi H., Tian J., Sun L., Hu X., Cui W., Du D. (2019). P2Y12 Regulates Microglia Activation and Excitatory Synaptic Transmission in Spinal Lamina II Neurons during Neuropathic Pain in Rodents. Cell Death Dis..

[134] Ochocka N., Kaminska B. (2021). Microglia Diversity in Healthy and Diseased Brain: Insights from Single-Cell Omics. Int. J. Mol. Sci..

[135] Jing F., Zhang Y., Long T., He W., Qin G., Zhang D., Chen L., Zhou J. (2019). P2Y12 Receptor Mediates Microglial Activation via RhoA/ROCK Pathway in the Trigeminal Nucleus Caudalis in a Mouse Model of Chronic Migraine. J. Neuroinflamm..

[136] Wei H., Zou H., Sheikh A.M., Malik M., Dobkin C., Brown W.T., Li X. (2011). IL-6 Is Increased in the Cerebellum of Autistic Brain and Alters Neural Cell Adhesion, Migration and Synaptic Formation. J. Neuroinflamm..

[137] Liu Y.U., Ying Y., Li Y., Eyo U.B., Chen T., Zheng J., Umpierre A.D., Zhu J., Bosco D.B., Dong H. (2019). Neuronal Network Activity Controls Microglial Process Surveillance in Awake Mice via Norepinephrine Signaling. Nat. Neurosci..

[138] Matyash M., Zabiegalov O., Wendt S., Matyash V., Kettenmann H. (2017). The Adenosine Generating Enzymes CD39/CD73 Control Microglial Processes Ramification in the Mouse Brain. PLoS ONE.

[139] Mellios N., Woodson J., Garcia R.I., Crawford B., Sharma J., Sheridan S.D., Haggarty S.J., Sur M. (2014). Β2-Adrenergic Receptor Agonist Ameliorates Phenotypes and Corrects MicroRNA-Mediated IGF1 Deficits in a Mouse Model of Rett Syndrome. Proc. Natl. Acad. Sci. USA.

[140] Connors S.L., Crowell D.E., Eberhart C.G., Copeland J., Newschaffer C.J., Spence S.J., Zimmerman A.W. (2005). β 2-Adrenergic Receptor Activation and Genetic Polymorphisms in Autism: Data from Dizygotic Twins. J. Child. Neurol..

[141] Masino S.A., Kawamura M., Plotkin L.M., Svedova J., DiMario F.J., Eigsti I.-M. (2011). The Relationship between the Neuromodulator Adenosine and Behavioral Symptoms of Autism. Neurosci. Lett.

[142] Lecchi A., Femia E., Paoletta S., Dupuis A., Ohlmann P., Gachet C., Jacobson K., Machura K., Podda G., Zieger B. (2016). Inherited Dysfunctional Platelet P2Y12 Receptor Mutations Associated with Bleeding Disorders. Hamostaseologie.

[143] Yang J., Yu Q., Xu Z., Zheng N., Zhong J., Li J., Liu Y., Xu H., Su J., Ji L. (2021). Clopidogrel Resistance Is Associated with DNA Methylation of Genes from Whole Blood of Humans. Front. Genet..

[144] Gidal B., Spencer N., Maly M., Pitterle M., Williams E., Collins M., Jones J. (1994). Valproate-mediated Disturbances of Hemostasis. Neurology.

[145] Naviaux R.K., Curtis B., Li K., Naviaux J.C., Bright A.T., Reiner G.E., Westerfield M., Goh S., Alaynick W.A., Wang L. (2017). Low-Dose Suramin in Autism Spectrum Disorder: A Small, Phase I/II, Randomized Clinical Trial. Ann. Clin. Transl. Neurol..

[146] Burnstock G. (2018). The Therapeutic Potential of Purinergic Signalling. Biochem. Pharm..

[147] Han S., Suzuki-Kerr H., Vlajkovic S.M., Thorne P.R. (2022). The Developmental Journey of Therapies Targeting Purine Receptors: From Basic Science to Clinical Trials. Purinergic Signal..

