# Correction: Babiec et al. Alterations of Purinergic Receptors Levels and Their Involvement in the Glial Cell Morphology in a Pre-Clinical Model of Autism Spectrum Disorders. *Brain Sci.* 2023, *13*, 1088

**DOI:** 10.3390/brainsci14030233

**Published:** 2024-02-28

**Authors:** Lidia Babiec, Anna Wilkaniec, Marta Matuszewska, Ewelina Pałasz, Magdalena Cieślik, Agata Adamczyk

**Affiliations:** Department of Cellular Signalling, Mossakowski Medical Research Institute, Polish Academy of Sciences, Pawińskiego 5, 02-106 Warsaw, Poland; lbabiec@imdik.pan.pl (L.B.); mmatuszewska@imdik.pan.pl (M.M.); epalasz@imdik.pan.pl (E.P.); mcieslik@imdik.pan.pl (M.C.); aadamczyk@imdik.pan.pl (A.A.)

## Figure Legend

In the original publication [1], there was a mistake in the legend for Scheme 1: (d) To analyse the modulation of purinergic signalling, rats at 52 PND were divided into three groups and subsequently injected with PPADS (P2X receptor antagonist), isoPPADS (250 mg/kg, i.p.) (P2 receptor antagonist), or saline. The correct legend appears below.

**Scheme 1.** Experimental design. (**a**) The analysis of changes in the levels of specific purinergic receptors during rat brain development was conducted at three stages: ED 19, 25 PND, and 52 PND. (**b**) A Valproic acid-induced model of ASD was performed by a single intraperitoneal injection of VPA (450 mg/kg, i.p.) or saline into pregnant rats. (**c**) The investigation of VPA-induced alteration on the expression of selected purinergic receptors was performed on the brains of 52-day-old animals. (**d**) To analyse the modulation of purinergic signalling, rats at 52 PND were divided into three groups and subsequently injected with PPADS (P2X receptor antagonist), isoPPADS (12.5 mg/kg, i.p.) (P2 receptor antagonist), or saline. Analysis of glial cells and inflammatory cytokine expression was performed on the brains of 54-day-old animals. The figure was created using BioRender.com

## Text Correction

There was an error in the original publication. The rats received a single intraperitoneal injection of either PPADS or isoPPADS at a dose of 250 mg/kg of body.

A correction has been made to Section 2.2:

The rats received a single intraperitoneal injection of either PPADS or isoPPADS at a dose of 12.5 mg/kg of body weight. Control rats received the appropriate volume of the vehicle.

The authors state that the scientific conclusions are unaffected. This correction was approved by the Academic Editor. The original publication has also been updated.
