# Supplementary material for: Alterations of Purinergic Receptors Levels and Their Involvement in the Glial Cell Morphology in a Pre-Clinical Model of Autism Spectrum Disorders
Source: Brain Sci. 2023 Jul 18;13(7):1088. doi: 10.3390/brainsci13071088 (PMC10377192; doi:10.3390/brainsci13071088)
Supplement: Supplementary file 1 [file brainsci-13-01088-s001.zip › supplementary materials.pptx]

## Slide 1
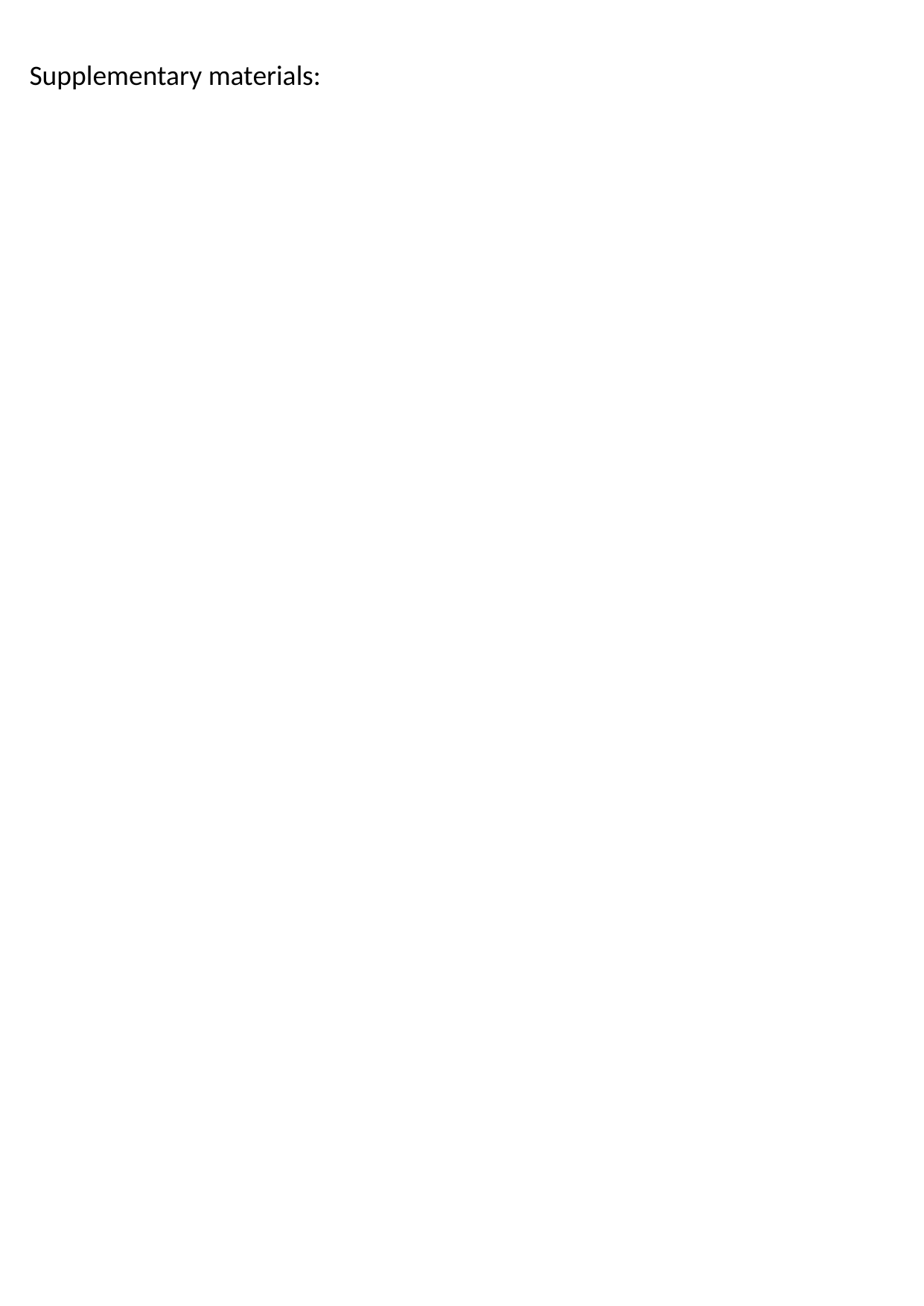

Supplementary materials:

## Slide 2
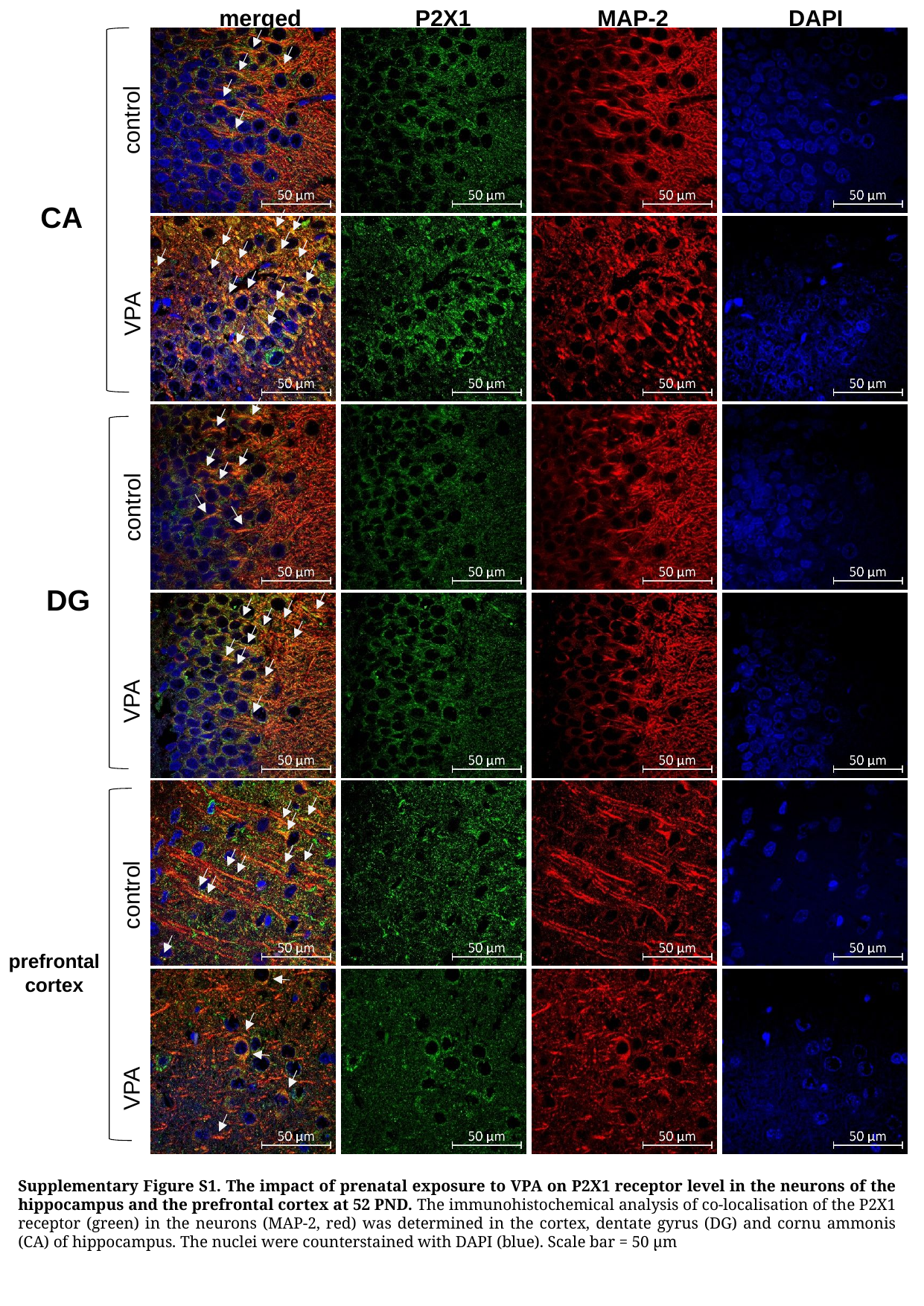

merged P2X1 MAP-2 DAPI
control
CA
VPA
control
DG
VPA
control
prefrontal cortex
VPA
Supplementary Figure S1. The impact of prenatal exposure to VPA on P2X1 receptor level in the neurons of the hippocampus and the prefrontal cortex at 52 PND. The immunohistochemical analysis of co-localisation of the P2X1 receptor (green) in the neurons (MAP-2, red) was determined in the cortex, dentate gyrus (DG) and cornu ammonis (CA) of hippocampus. The nuclei were counterstained with DAPI (blue). Scale bar = 50 μm

## Slide 3
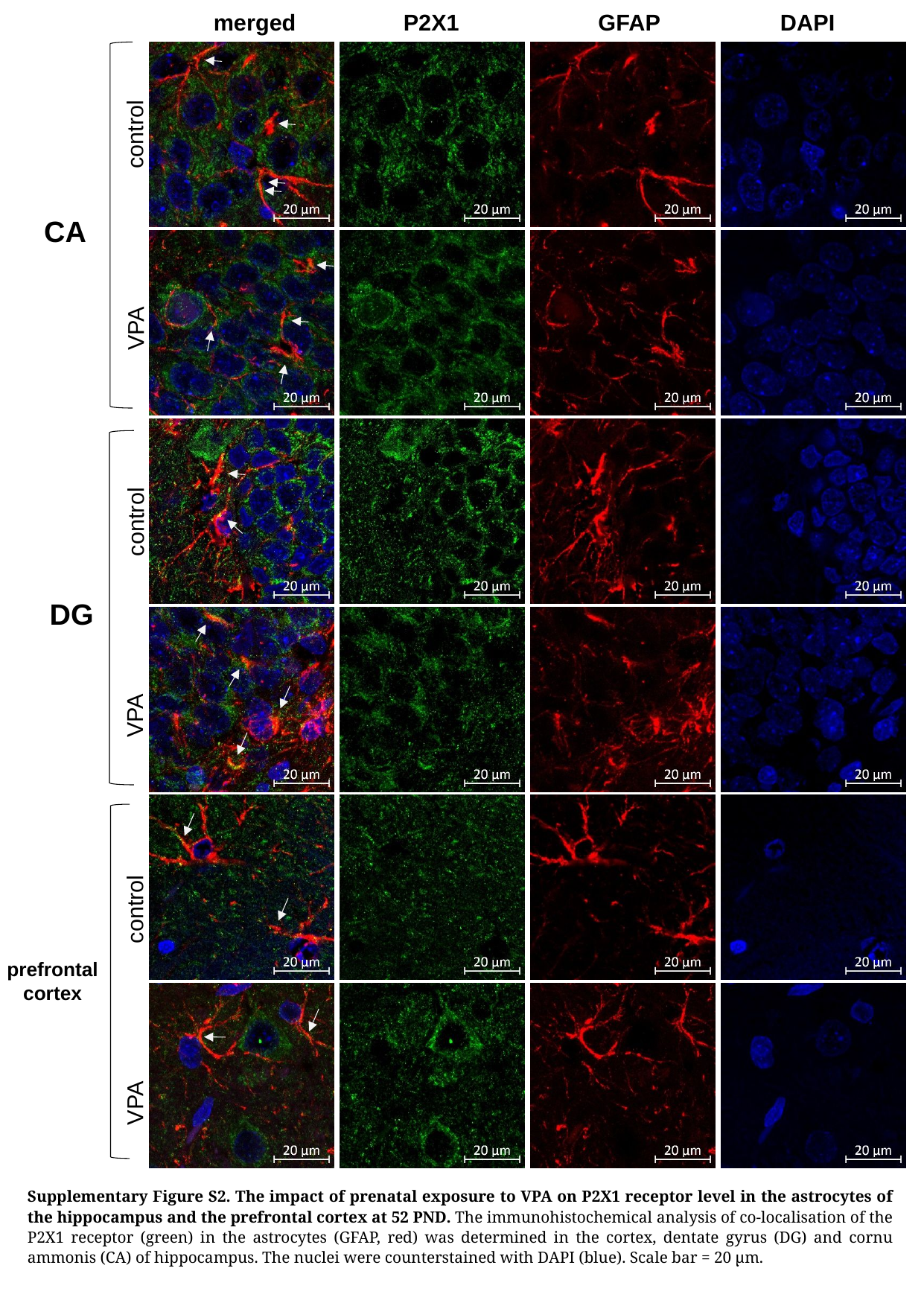

merged P2X1 GFAP DAPI
control
CA
VPA
control
DG
VPA
control
prefrontal cortex
VPA
Supplementary Figure S2. The impact of prenatal exposure to VPA on P2X1 receptor level in the astrocytes of the hippocampus and the prefrontal cortex at 52 PND. The immunohistochemical analysis of co-localisation of the P2X1 receptor (green) in the astrocytes (GFAP, red) was determined in the cortex, dentate gyrus (DG) and cornu ammonis (CA) of hippocampus. The nuclei were counterstained with DAPI (blue). Scale bar = 20 μm.

## Slide 4
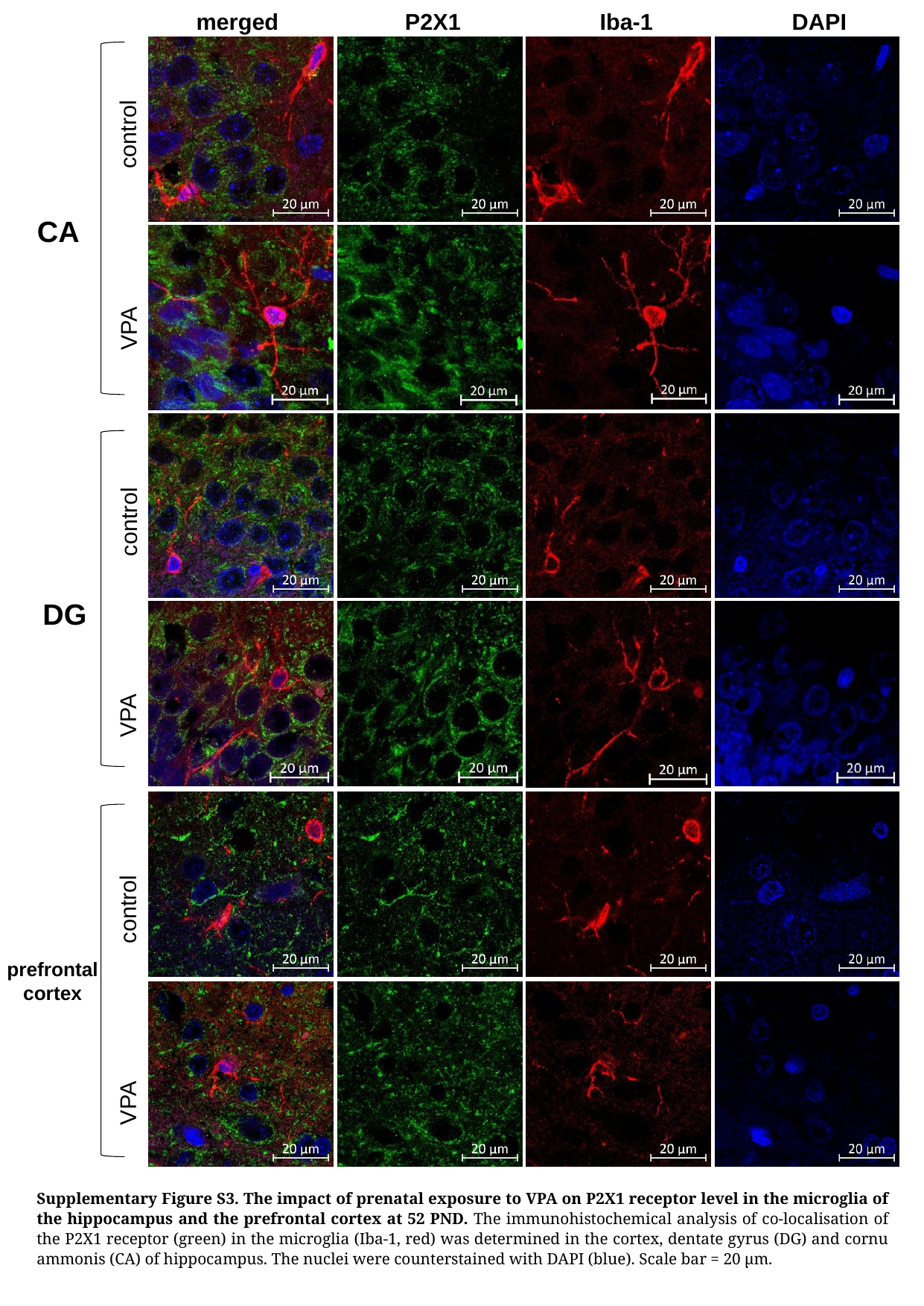

merged P2X1 Iba-1 DAPI
control
CA
VPA
control
DG
VPA
control
prefrontal cortex
VPA
Supplementary Figure S3. The impact of prenatal exposure to VPA on P2X1 receptor level in the microglia of the hippocampus and the prefrontal cortex at 52 PND. The immunohistochemical analysis of co-localisation of the P2X1 receptor (green) in the microglia (Iba-1, red) was determined in the cortex, dentate gyrus (DG) and cornu ammonis (CA) of hippocampus. The nuclei were counterstained with DAPI (blue). Scale bar = 20 μm.

## Slide 5
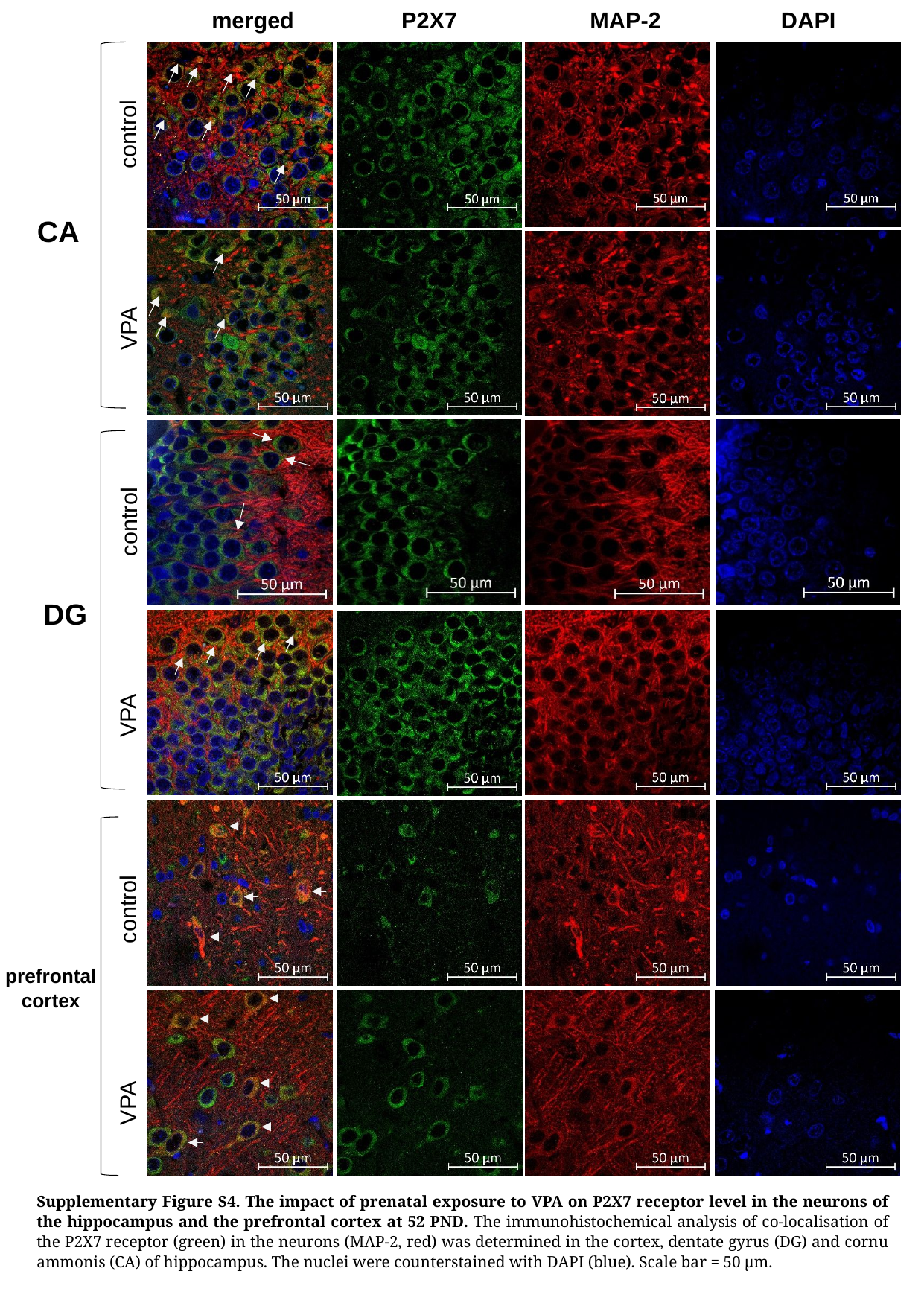

merged P2X7 MAP-2 DAPI
control
CA
VPA
control
DG
VPA
control
prefrontal cortex
VPA
Supplementary Figure S4. The impact of prenatal exposure to VPA on P2X7 receptor level in the neurons of the hippocampus and the prefrontal cortex at 52 PND. The immunohistochemical analysis of co-localisation of the P2X7 receptor (green) in the neurons (MAP-2, red) was determined in the cortex, dentate gyrus (DG) and cornu ammonis (CA) of hippocampus. The nuclei were counterstained with DAPI (blue). Scale bar = 50 μm.

## Slide 6
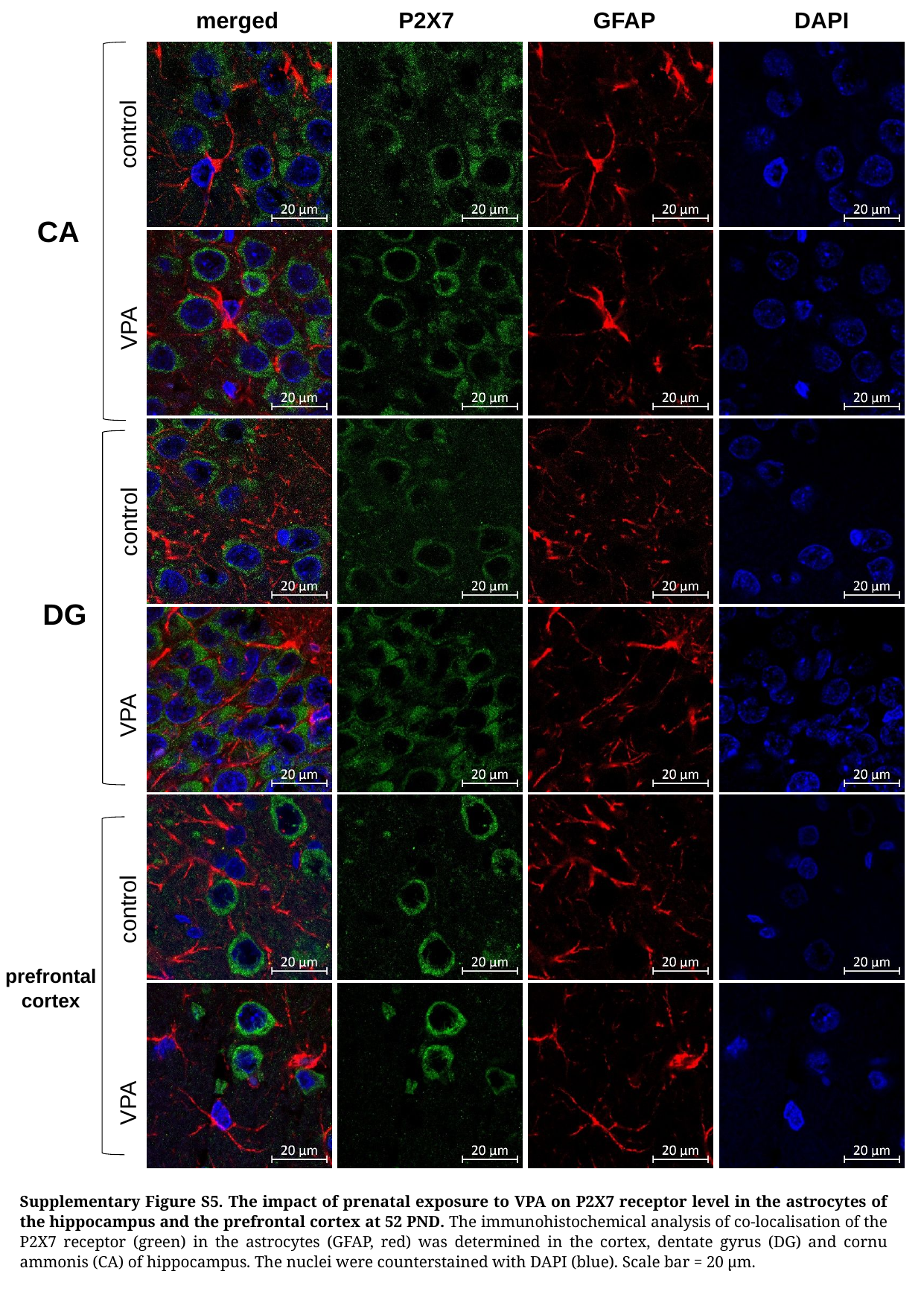

merged P2X7 GFAP DAPI
control
CA
VPA
control
DG
VPA
control
prefrontal cortex
VPA
Supplementary Figure S5. The impact of prenatal exposure to VPA on P2X7 receptor level in the astrocytes of the hippocampus and the prefrontal cortex at 52 PND. The immunohistochemical analysis of co-localisation of the P2X7 receptor (green) in the astrocytes (GFAP, red) was determined in the cortex, dentate gyrus (DG) and cornu ammonis (CA) of hippocampus. The nuclei were counterstained with DAPI (blue). Scale bar = 20 μm.

## Slide 7
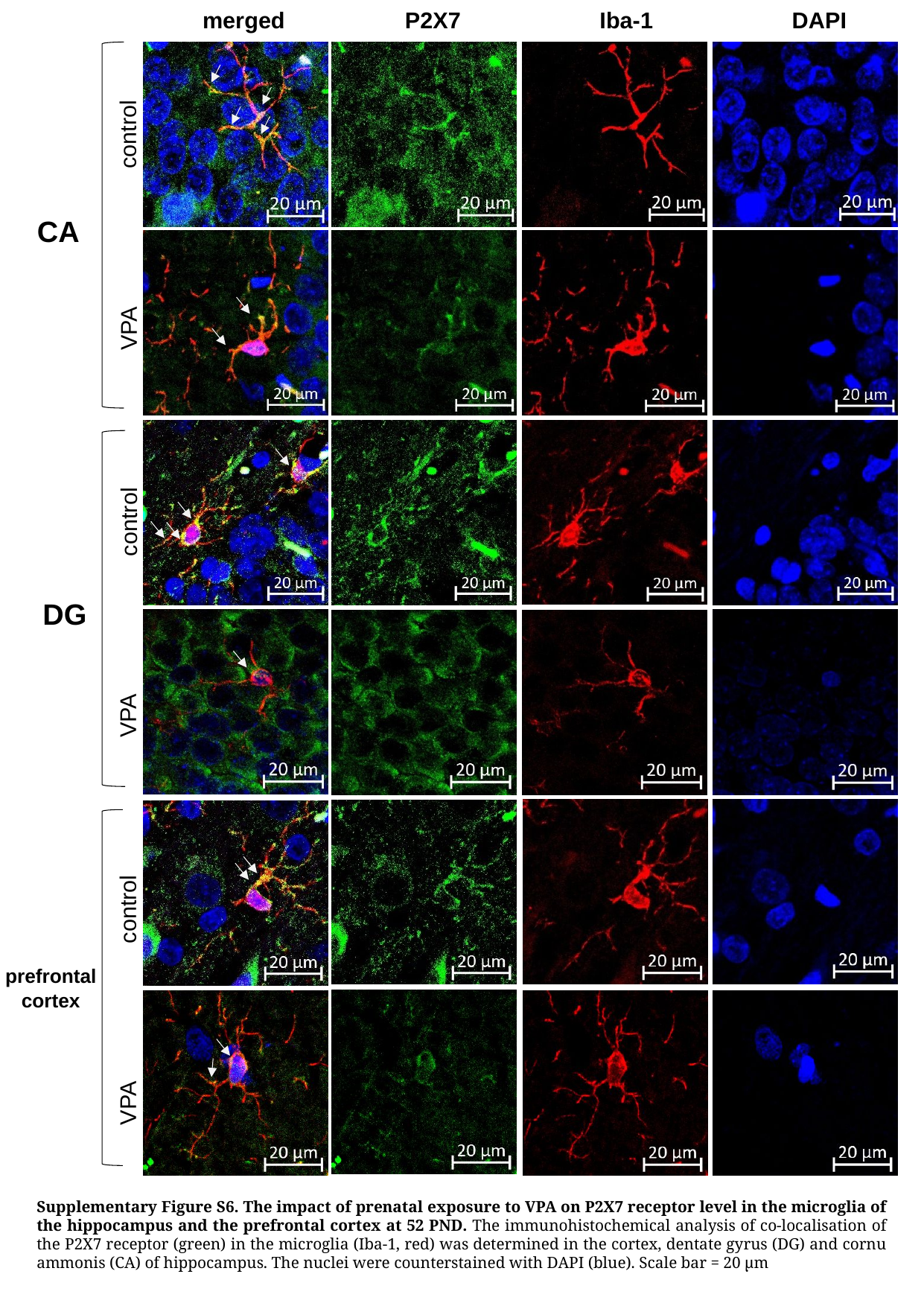

merged P2X7 Iba-1 DAPI
P2X7
control
CA
VPA
control
DG
VPA
control
prefrontal cortex
VPA
Supplementary Figure S6. The impact of prenatal exposure to VPA on P2X7 receptor level in the microglia of the hippocampus and the prefrontal cortex at 52 PND. The immunohistochemical analysis of co-localisation of the P2X7 receptor (green) in the microglia (Iba-1, red) was determined in the cortex, dentate gyrus (DG) and cornu ammonis (CA) of hippocampus. The nuclei were counterstained with DAPI (blue). Scale bar = 20 μm

## Slide 8
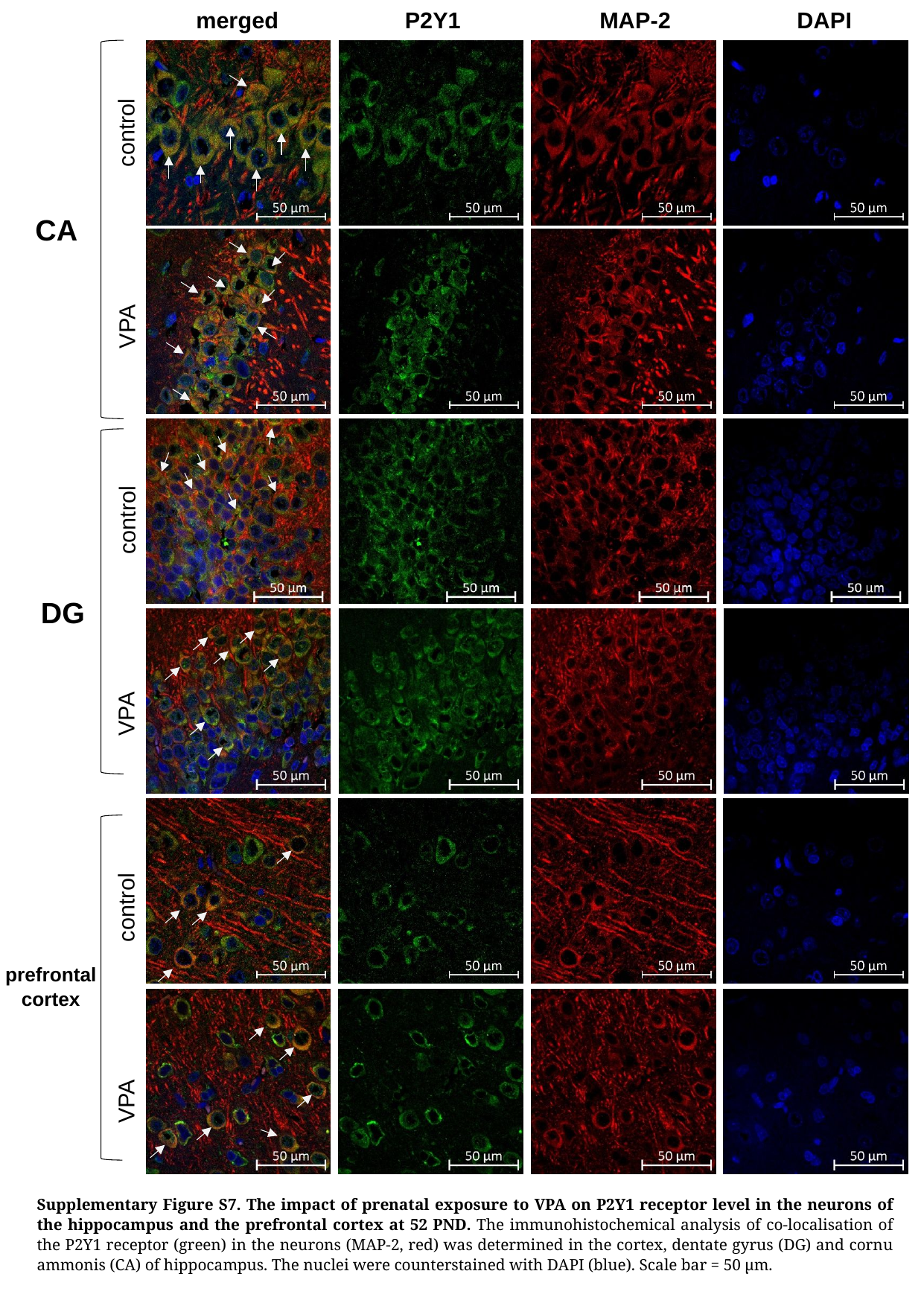

merged P2Y1 MAP-2 DAPI
control
CA
VPA
control
DG
VPA
control
prefrontal cortex
VPA
Supplementary Figure S7. The impact of prenatal exposure to VPA on P2Y1 receptor level in the neurons of the hippocampus and the prefrontal cortex at 52 PND. The immunohistochemical analysis of co-localisation of the P2Y1 receptor (green) in the neurons (MAP-2, red) was determined in the cortex, dentate gyrus (DG) and cornu ammonis (CA) of hippocampus. The nuclei were counterstained with DAPI (blue). Scale bar = 50 μm.

## Slide 9
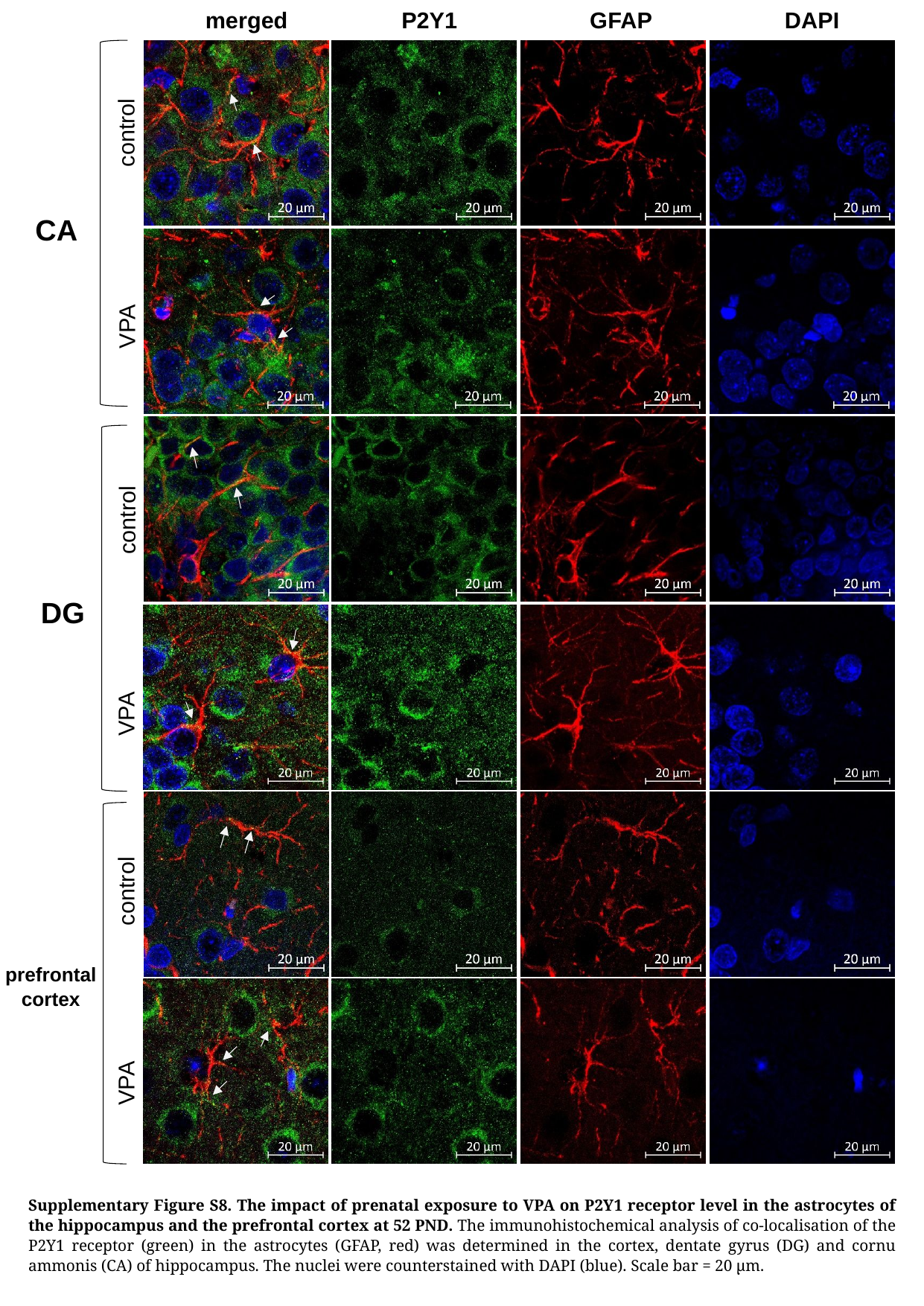

merged P2Y1 GFAP DAPI
P2Y1
control
CA
VPA
control
DG
VPA
control
prefrontal cortex
VPA
Supplementary Figure S8. The impact of prenatal exposure to VPA on P2Y1 receptor level in the astrocytes of the hippocampus and the prefrontal cortex at 52 PND. The immunohistochemical analysis of co-localisation of the P2Y1 receptor (green) in the astrocytes (GFAP, red) was determined in the cortex, dentate gyrus (DG) and cornu ammonis (CA) of hippocampus. The nuclei were counterstained with DAPI (blue). Scale bar = 20 μm.

## Slide 10
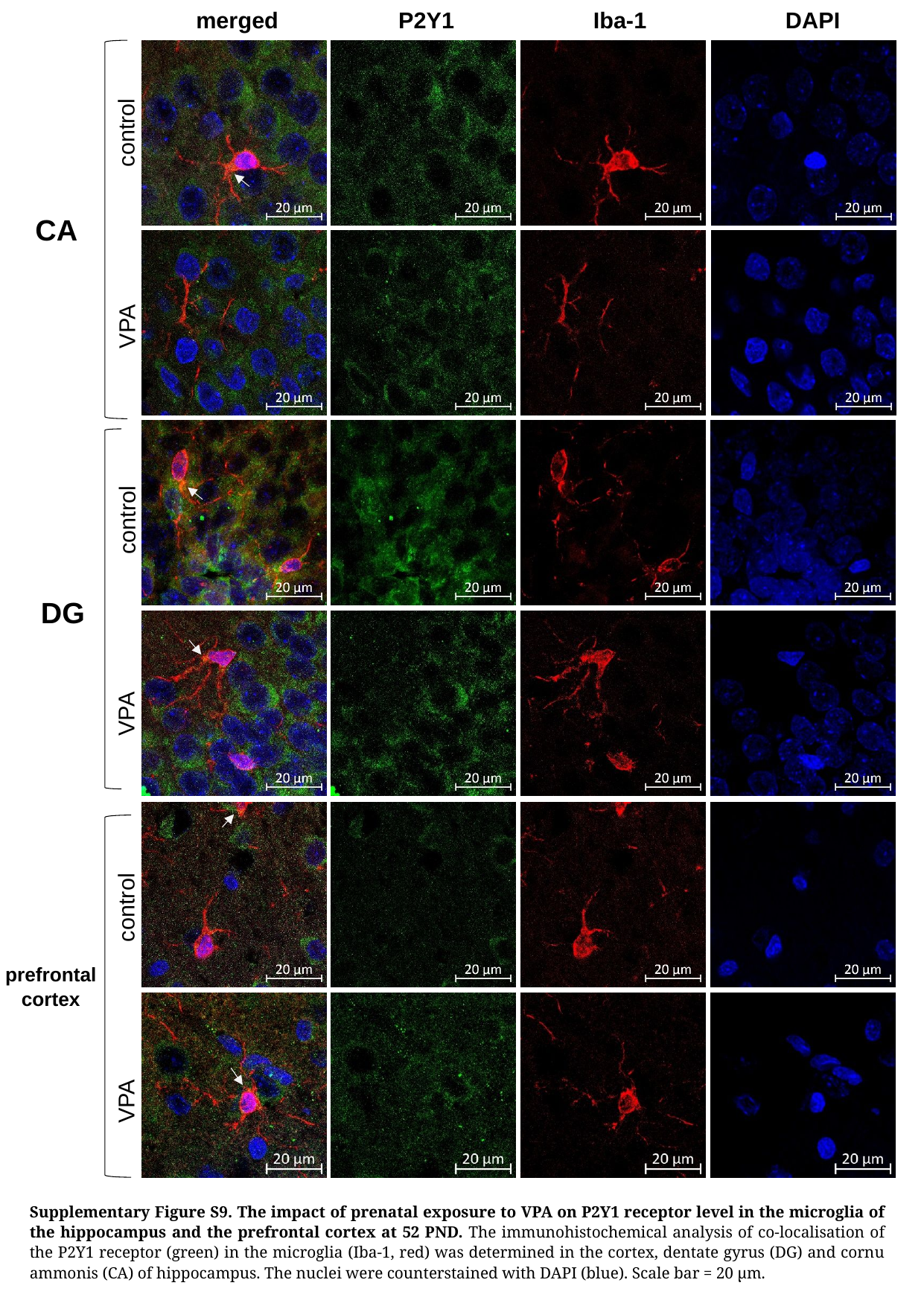

merged P2Y1 Iba-1 DAPI
P2Y1
control
CA
VPA
control
DG
VPA
control
prefrontal cortex
VPA
Supplementary Figure S9. The impact of prenatal exposure to VPA on P2Y1 receptor level in the microglia of the hippocampus and the prefrontal cortex at 52 PND. The immunohistochemical analysis of co-localisation of the P2Y1 receptor (green) in the microglia (Iba-1, red) was determined in the cortex, dentate gyrus (DG) and cornu ammonis (CA) of hippocampus. The nuclei were counterstained with DAPI (blue). Scale bar = 20 μm.

## Slide 11
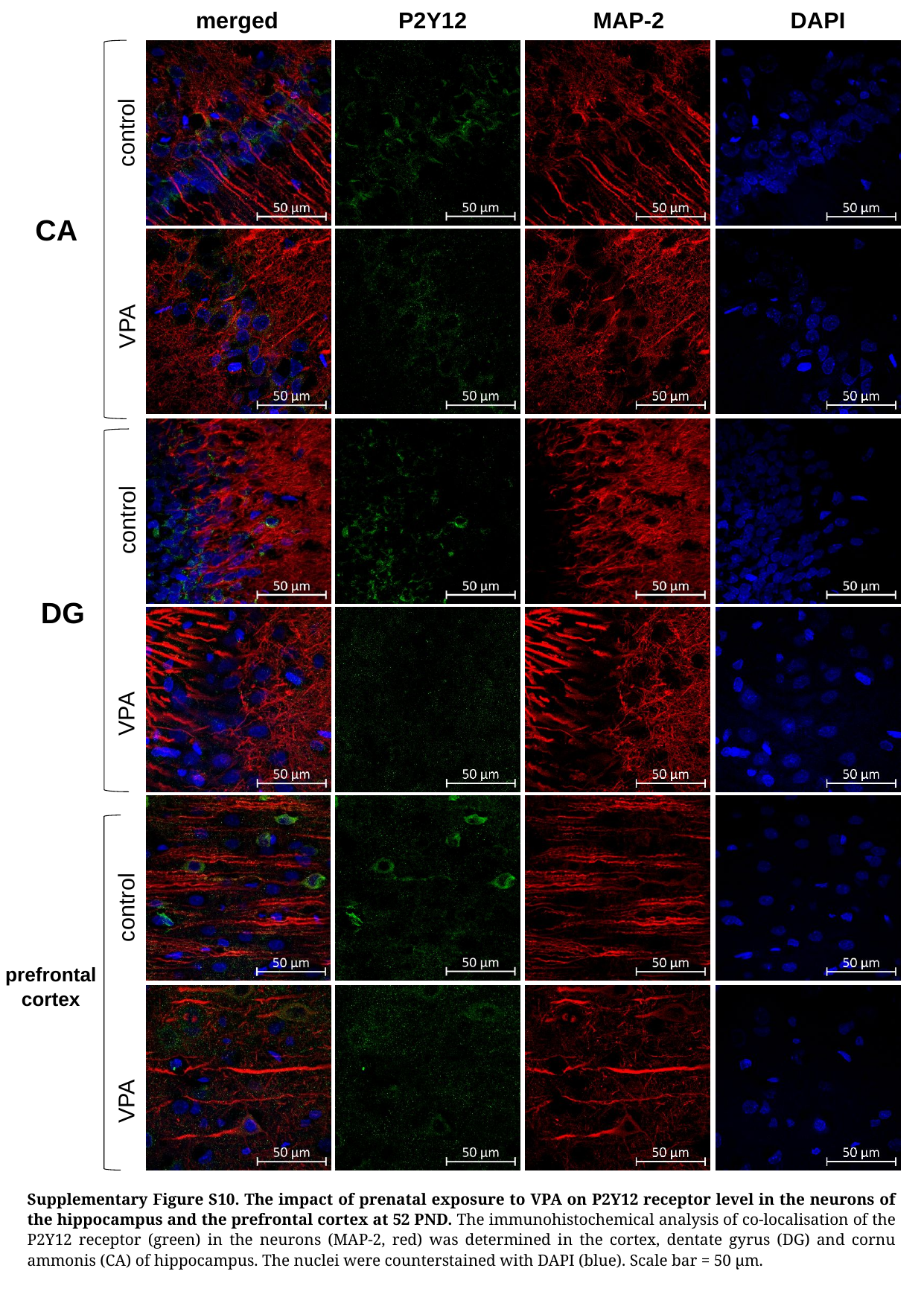

merged P2Y12 MAP-2 DAPI
control
CA
VPA
control
DG
VPA
control
prefrontal cortex
VPA
Supplementary Figure S10. The impact of prenatal exposure to VPA on P2Y12 receptor level in the neurons of the hippocampus and the prefrontal cortex at 52 PND. The immunohistochemical analysis of co-localisation of the P2Y12 receptor (green) in the neurons (MAP-2, red) was determined in the cortex, dentate gyrus (DG) and cornu ammonis (CA) of hippocampus. The nuclei were counterstained with DAPI (blue). Scale bar = 50 μm.

## Slide 12
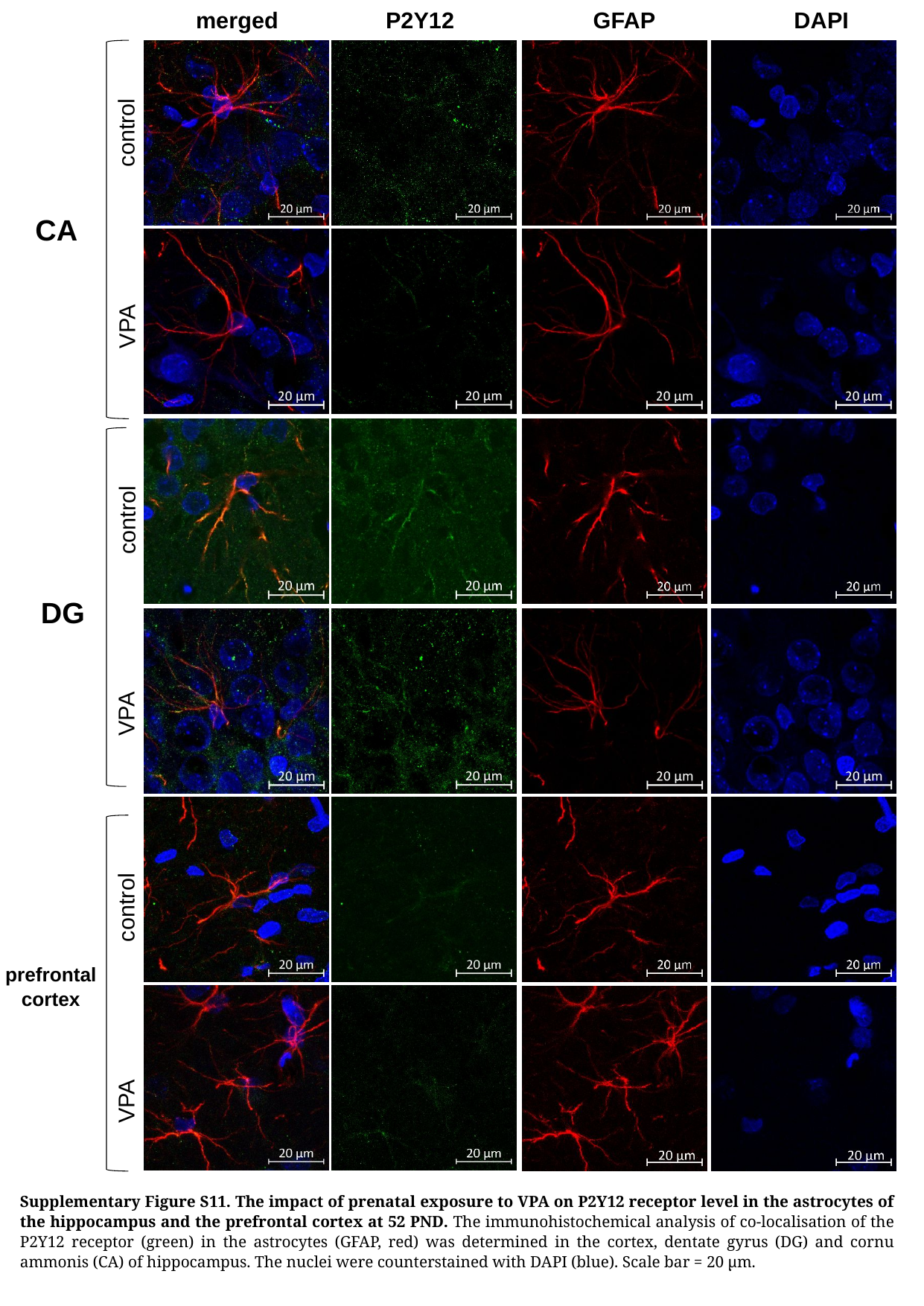

merged P2Y12 GFAP DAPI
P2Y1
control
CA
VPA
control
DG
VPA
control
prefrontal cortex
VPA
Supplementary Figure S11. The impact of prenatal exposure to VPA on P2Y12 receptor level in the astrocytes of the hippocampus and the prefrontal cortex at 52 PND. The immunohistochemical analysis of co-localisation of the P2Y12 receptor (green) in the astrocytes (GFAP, red) was determined in the cortex, dentate gyrus (DG) and cornu ammonis (CA) of hippocampus. The nuclei were counterstained with DAPI (blue). Scale bar = 20 μm.

## Slide 13
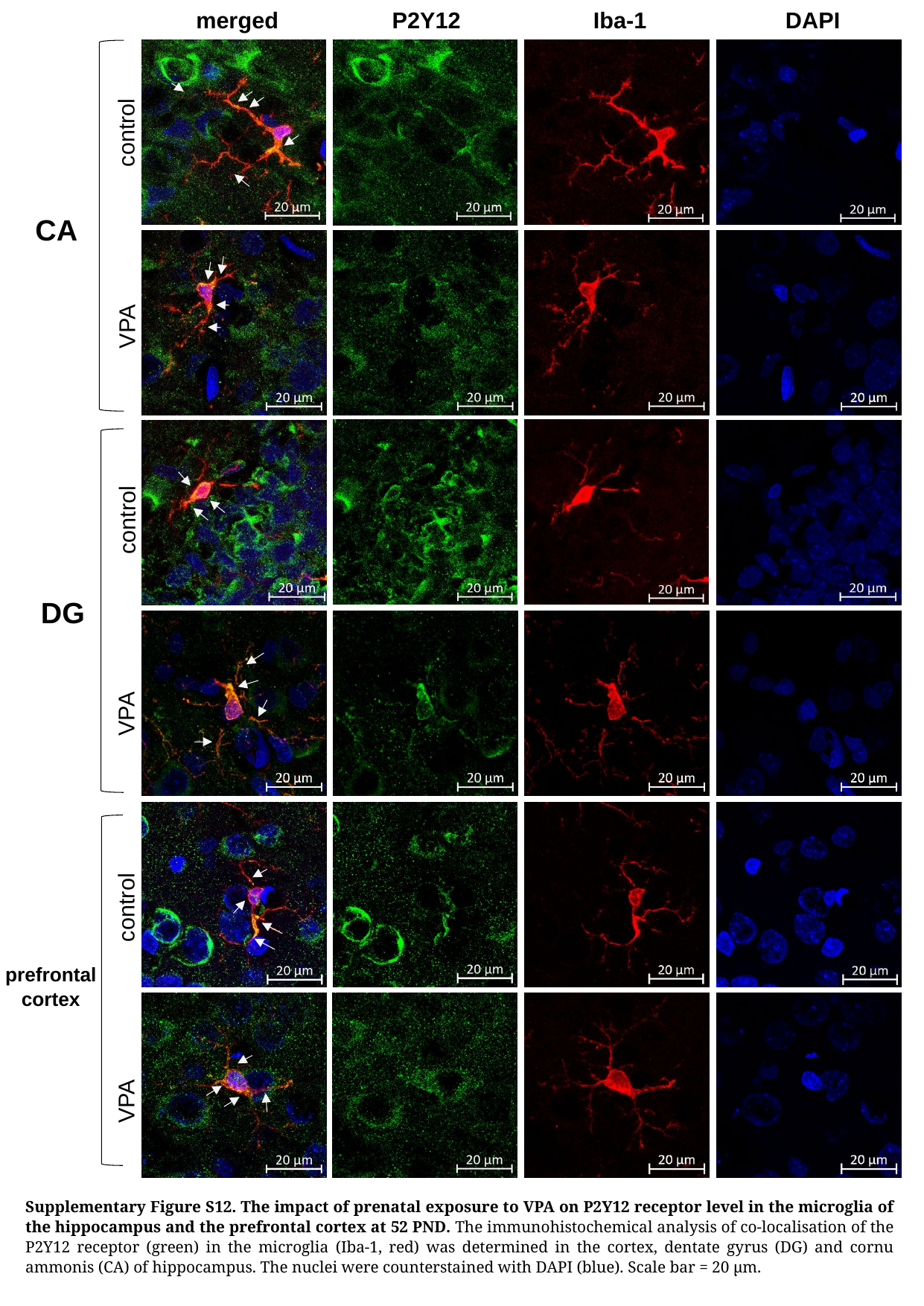

merged P2Y12 Iba-1 DAPI
P2Y1
control
CA
VPA
control
DG
VPA
control
prefrontal cortex
VPA
Supplementary Figure S12. The impact of prenatal exposure to VPA on P2Y12 receptor level in the microglia of the hippocampus and the prefrontal cortex at 52 PND. The immunohistochemical analysis of co-localisation of the P2Y12 receptor (green) in the microglia (Iba-1, red) was determined in the cortex, dentate gyrus (DG) and cornu ammonis (CA) of hippocampus. The nuclei were counterstained with DAPI (blue). Scale bar = 20 μm.

## Slide 14
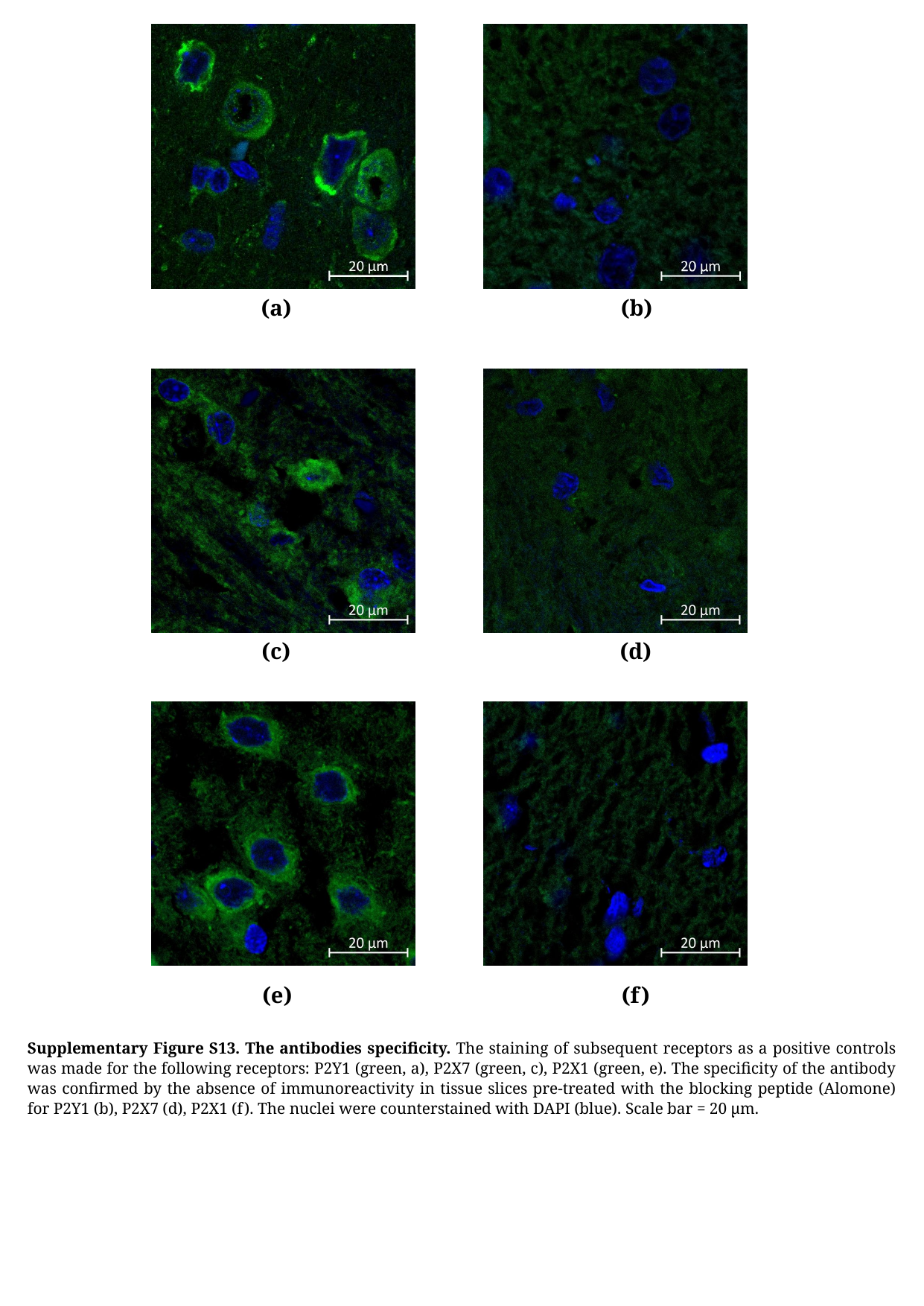

(a) (b)
(c) (d)
(e) (f)
Supplementary Figure S13. The antibodies specificity. The staining of subsequent receptors as a positive controls was made for the following receptors: P2Y1 (green, a), P2X7 (green, c), P2X1 (green, e). The specificity of the antibody was confirmed by the absence of immunoreactivity in tissue slices pre-treated with the blocking peptide (Alomone) for P2Y1 (b), P2X7 (d), P2X1 (f). The nuclei were counterstained with DAPI (blue). Scale bar = 20 μm.
